# Highly Porous Polymer Beads Coated with Nanometer-Thick Metal Oxide Films for Photocatalytic Oxidation of Bisphenol A

**DOI:** 10.1021/acsanm.3c03891

**Published:** 2023-10-24

**Authors:** Gergő Ballai, Tomaž Kotnik, Matjaž Finšgar, Albin Pintar, Zoltán Kónya, András Sápi, Sebastijan Kovačič

**Affiliations:** † Interdisciplinary Excellence Centre, Department of Applied and Environmental Chemistry, University of Szeged, Rerrich Béla tér 1, H-6720 Szeged, Hungary; ‡ Department of Inorganic Chemistry and Technology, National Institute of Chemistry, Hajdrihova 19, SI-1001 Ljubljana, Slovenia; § Faculty of Chemistry and Chemical Technology, University of Ljubljana, Večna Pot 113, 1000 Ljubljana, Slovenia; ∥ University of Maribor, Faculty of Chemistry and Chemical Engineering, Smetanova 17, SI-2000 Maribor, Slovenia; ⊥ MTA-SZTE Reaction Kinetics and Surface Chemistry Research Group, Rerrich Béla tér 1, H-6720 Szeged, Hungary

**Keywords:** emulsion-templating, macroporous polymers, atomic-layer-deposition, metal-oxide, nanocoating, heterogeneous photocatalysis

## Abstract

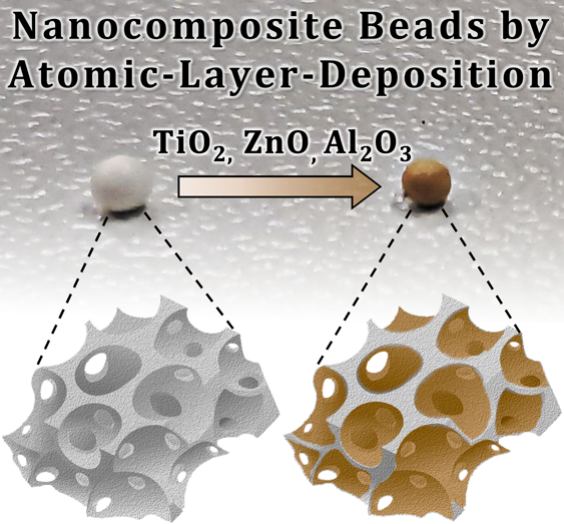

Highly porous metal oxide–polymer nanocomposites are attracting considerable interest due to their unique structural and functional features. A porous polymer matrix brings properties such as high porosity and permeability, while the metal oxide phase adds functionality. For the metal oxide phase to perform its function, it must be fully accessible, and this is possible only at the pore surface, but functioning surfaces require controlled engineering, which remains a challenge. Here, highly porous nanocomposite beads based on thin metal oxide nanocoatings and polymerized high internal phase emulsions (polyHIPEs) are demonstrated. By leveraging the unique properties of polyHIPEs, i.e., a three-dimensional (3D) interconnected network of macropores, and high-precision of the atomic-layer-deposition technique (ALD), we were able to homogeneously coat the entire surface of the pores in polyHIPE beads with TiO_2_-, ZnO-, and Al_2_O_3_-based nanocoatings. Parameters such as nanocoating thickness, growth per cycle (GPC), and metal oxide (MO) composition were systematically controlled by varying the number of deposition cycles and dosing time under specific process conditions. The combination of polyHIPE structure and ALD technique proved advantageous, as MO-nanocoatings with thicknesses between 11 ± 3 and 40 ± 9 nm for TiO_2_ or 31 ± 6 and 74 ± 28 nm for ZnO and Al_2_O_3_, respectively, were successfully fabricated. It has been shown that the number of ALD cycles affects both the thickness and crystallinity of the MO nanocoatings. Finally, the potential of ALD-derived TiO_2_-polyHIPE beads in photocatalytic oxidation of an aqueous bisphenol A (BPA) solution was demonstrated. The beads exhibited about five times higher activity than nanocomposite beads prepared by the conventional (Pickering) method. Such ALD-derived polyHIPE nanocomposites could find wide application in nanotechnology, sensor development, or catalysis.

## 1 Introduction

The idea of compounding metal oxides (MO) into porous polymer matrices is not new, as numerous MO–polymer pairs and a variety of approaches to create porous structures have been combined over the past decades to produce porous polymer nanocomposites.^1,2^ Approaches for preparing porous polymer nanocomposites include the sol–gel method, electrospinning, chemical vapor deposition (CVD), nanocasting, or template-assisted synthesis, to name a few; however, the choice of a particular synthesis method depends on what specific properties we want to tune/control in a porous nanocomposite, either the porous structure, the chemical composition, or the spatial distribution of nanoparticles.^3−5^

Among the approaches to generate porous polymer matrices, polymerization of high internal phase emulsion templates (referred to as polyHIPEs) has lately gained particular attention.^6,7^ The reason for its popularity is that both the chemistry of the polymer backbone and the porous properties, i.e., pore volume, pore size distribution, and degree of three-dimensional (3D) interconnectivity, can be easily tuned and controlled.^8^ The polyHIPE (PH) matrices are also very useful for obtaining porous nanocomposites and hybrids.^9,10^ While early efforts focused primarily on improving the mechanical properties of PH matrices,^11−13^ more recent examples increasingly aim to add functions to PH such as conductive,^14−17^ magnetic,^18−21^ thermo-insulative,^22,23^ catalytic,^24−27^ or capture properties,^28−31^ to name a few, by obtaining PH nanocomposites. A number of methods have been developed to incorporate MO into PHs, which can be divided into two main strategies. The first is to impregnate the surface of the voids in the preformed PH with a solution containing the precursor of the desired MO.^32−34^ A second option is to use preformed MO as stabilizers for HIPEs, a process known as Pickering-stabilization, in which MO remains embedded in or attached to the surface of the voids after polymerization.^35^ However, both strategies are associated with difficulties, such as aggregation-related defects, homogeneity, lack of control over the location of the NPs (surface vs bulk phase), batch-to-batch reproducibility, and, in some cases, even clogging of the interconnecting pores by the larger NP agglomerates. Therefore, the preparation of functional PH nanocomposites still remains a challenging task, as efficient hybridization is required to achieve synergy of the components at the nanocomposite interface; otherwise, it is merely a physical mixing. In response to the shortcomings of the above strategies, in this work, we present a different approach to obtain functional MO-PH nanocomposites using atomic-layer deposition (ALD).

ALD is a thin-film synthesis technique that is particularly well-suited for the deposition of a variety of materials on porous matrices. In the ALD process, the solid (porous) matrix is exposed in alternating cycles to vaporized chemical precursors that react with functional groups on the pore surface and deposit the material in a layer-by-layer fashion.^36,37^ Since a chemical reaction ends when all functional groups on the pore surface have reacted with the gaseous precursors, ALD is a self-limiting process that allows precise film thickness control.^38,39^ However, for an efficient ALD process, two main conditions must be met; i.e., an interconnected porous structure and the matrix surface should have a high density of reactive surface sites.^40,41^ When the PH matrix is used in ALD, its foamy structure with 3D-interconnectivity should be advantageous for facilitating the diffusion of the precursor vapor molecules into the porous structure, reaching the available reactive sites on the void surface. Another favorable feature is a broad selection of monomeric systems from which PH can be synthesized, which, in combination with the possible chemical functionalization after polymerization,^42,43^ allows the preparation of a tailored surface-chemistry required for successful ALD. More than a decade ago, Weimer et al. reported the fabrication of ceramic or biocompatible interfaces using a relatively nonreactive and hydrophobic styrene-based PH as a porous matrix for ALD.^44,45^ The combination of PH matrices and ALD holds great potential for the development of porous nanocomposites with well-defined (nano)functionalized surfaces. Thus, in this work, reactive and hydrophilic poly(acrylamide) (PAM) PH beads are used as an example and different MOs are synthesized on the surface of the voids. Of particular interest is the ability to form MO nanocoatings exclusively on the surface with precise control of thickness and complete accessibility. Finally, we discuss the synthesis–structure relationship of TiO_2_-, Al_2_O_3_-, and ZnO-nanocoated PH beads and evaluate the photocatalytic properties during the oxidation of the endocrine disrupting compound bisphenol A (BPA) dissolved in water.

## 2 Experimental Section

### Materials

Methylenebis(acrylamide) (MBAA, Sigma), *N*,*N*,*N*′,*N*′-tetramethyl ethylenediamine (TMEDA, Merck), Pluronic F127 (F127, Sigma), acrylamide (AAM, Sigma), ammonium persulfate (APS, Sigma-Aldrich), toluene for analysis (Merck), and paraffin oil (density at 20 °C 0.865 kg/L, Pharmachem) were all used as received. For the atomic layer deposition, three precursors were used, titanium tetrachloride (Thermo Fisher Scientific, 99.0%), diethylzinc (STREM, 99.9998% Zn), trimethylaluminum (STREM, 99.999% Al), and the oxidizer was ultrapure water (obtained from Milli-Q purification system) in all cases.

### Preparation of Poly(acrylamide) (PAAM)-Based polyHIPE Beads through O/W/O Sedimentation Polymerization

**Scheme 1:**
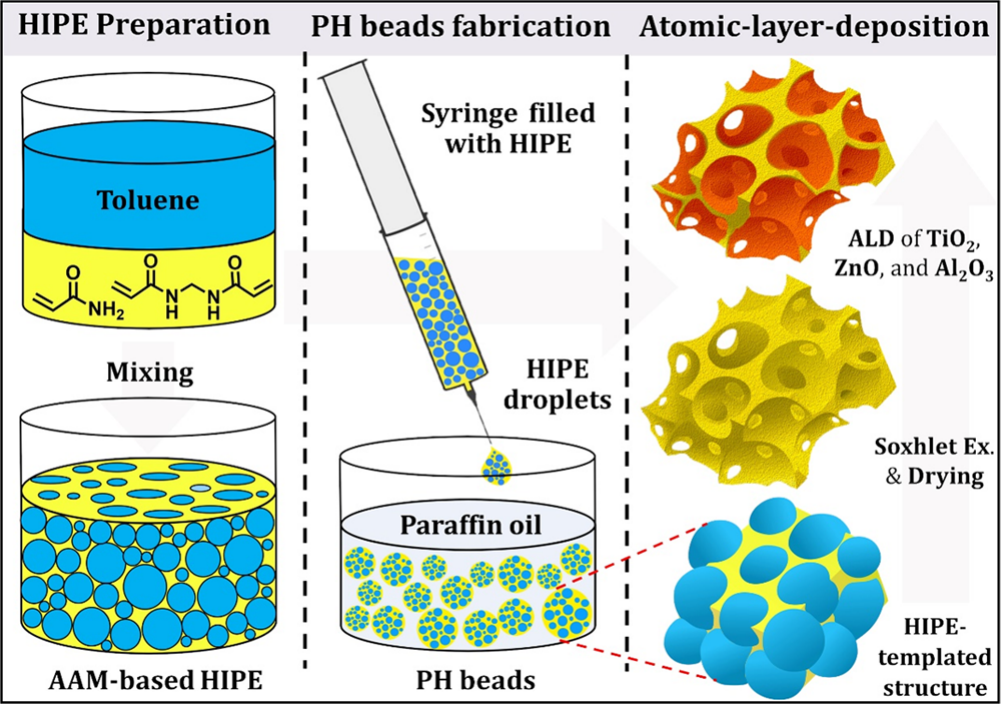
Schematic Presentation of the Bead Synthesis and MO-Coating via ALD.

O/W HIPEs were formed by adding toluene as an organic internal phase dropwise to the aqueous external phase, consisting of AAM (monomers), MBAA (cross-linking comonomer), initiator (APS), and surfactant (Pluronic F-108) during continuous stirring (masses according to the Table S1). Each reagent was dissolved completely before the addition of the next one. After the addition of the toluene containing TMEDA, the O/W HIPE was further stirred for 5 min. Then the O/W HIPE was injected dropwise into the second continuous phase (paraffin oil) also containing TMEDA, using a syringe with a needle (external diameter of 0.8 mm) (Scheme 1). 40 mL of paraffin oil was charged into a 50 mL graduated cylinder, degassed in an ultrasonic bath, and then heated in a water bath to 85 °C. After whole O/W HIPE has been added dropwise, the graduated cylinder was allowed to stand in the water bath for 1 h and then transferred to an oven at 50 °C for 24 h to complete polymerization. After polymerization, beads were collected and cleaned in a Soxhlet apparatus for 24 h with ethanol and 24 h with ether and dried in a vacuum.

### ALD of MO in the PHs

PAAM-based PH beads were coated by TiO_2_, Al_2_O_3_, and ZnO films by Beneq TFS 200 Atomic Layer Deposition equipment using titanium tetrachloride (TiCl_4_), diethylzinc (Zn(C_2_H_5_)_2_; DEZ), or trimethylaluminum (Al(CH_3_)_3_; TMA) as precursor molecules, respectively. Throughout the deposition, the high aspect ratio (HAR) chamber was used. The dosing times of precursor molecules were 10 s for both Al_2_O_3_ and ZnO, while in the case of TiO_2_ it was 10, 30, 60, and 100 s. The deposition temperatures were 150 °C in the case of Al_2_O_3_, 175 °C in the case of ZnO, and 250 °C in the case of TiO_2_, respectively. The resulting materials are termed MO-X-Y, where MO refers to the metal oxide used, X to the number of coating cycles, and Y to the dosing time.

### TiO_2_-Pickering PH Bead

TiO_2_-PAAM polyHIPE beads were prepared by the conventional Pickering HIPE-templating method. First, oil-in-water (O/W) HIPEs were prepared using TiO_2_ and Pluronic F-108 as costabilizers, and then beads were formed by O/W/O sedimentation polymerization (see details in Supporting Information (SI)).

### Characterization

TGA measurements were performed with a TA Instruments Q500 Thermogravimetric Analyzer (crucibles: 100 μL of platinum from TA Instruments). An air flow of 60 mL/min was used, and the heating rate until a final temperature of 800 °C was 10 °C/min. Morphology investigations were performed by scanning electron microscopy (SEM) using a Thermo Scientific Apreo S. microscope. Micrographs were taken at several magnifications from 1000- to 20000-fold, at 11–12 mm working distance, and at 10 kV acceleration voltage. The samples were broken and mounted on a carbon tab, and a thin layer of gold was sputtered onto the samples. The ETD detector of the microscope was used. The elemental analysis and the energy dispersive X-ray spectroscopy (EDS) mapping of the samples were performed by a Bruker Quantax EDS built into the Thermo Scientific Apreo S electron microscope. The line profile analysis was performed with the same instrument over the diameter of the bisected PH bead. For TEM analysis, samples were embedded in Sigma Epoxy embedding mixture, which was composed of 45.5% w/w epoxy embedding medium solution, 28.5% w/w hardener DDSA, 24.5% w/w NMA solution, and 1.5% w/w accelerator DMP-30. Infiltration was performed stepwise (impregnation at room temperature for 24 h and polymerization at 60 °C for 48 h). The resin embedded specimens were mounted in special holders, which at the same time fit the microtome. Ultrathin sections (50–100 nm) were obtained by using a Leica EM UC7 ultramicrotome (Leica, Wetzlar, Germany) equipped with a diamond knife (Diatome, Switzerland). Sections for TEM analysis were collected on 400-mesh copper TEM grids and examined in Technai G^2^ 20x Twin (Philips/FEI, Eindhoven) electron microscopes at 200 kV accelerating voltage. No staining has been applied. The X-ray diffractograms of the samples were recorded by a Rigaku Miniflex II diffractometer using Cu K_α_ radiation at a scan speed of 2° min^–1^ after comminuting the samples in an achate mortar. X-ray photoelectron spectroscopy (XPS) measurements were performed using a Supra+ device (Kratos, Manchester, UK) equipped with an Al K_α_ excitation source (additional information is given in SI).

## 3 Results and Discussion

### PHs Synthesis and Properties

In the first step, emulsion-templated PAAM beads were synthesized through the oil-in-water-in-oil (O/W/O) sedimentation polymerization.^46^ In practice, toluene-in-(AAm)aq (O/W) HIPE was first prepared and then injected as individual droplets with a syringe into a heated sedimentation medium (silicone/paraffin oil), forming an O/W/O double emulsion (Scheme 1). These droplets were polymerized by free radical polymerization within the external phase of the O/W HIPEs. After purification and drying, the PAAM PH beads had an average diameter of 1.5 ± 0.5 mm (Figure S1) and a total porosity of ∼90%, calculated from the PH density (ρ_PH_) of ∼0.09 g·cm^–3^ and the PAAM skeletal density (ρ_P_) of ∼0.920 g·cm^–3^. The most important, PAAM beads exhibited a typical 3D-interconnected microcellular PH morphology with an average void and window diameter of 10 ± 3 and 3 ± 1 μm, respectively (Figure S1). It was also confirmed that the voids were open to the bead surface. Finally, FTIR spectroscopy confirmed the molecular structure of the PAAM network cross-linked with MBAAM; typical stretching vibration peaks at 1645 cm^–1^ (amide C=O (amide I)), 1611 cm^–1^ (N–H bending), 3109 and 3340 cm^–1^ (N–H stretching), and 1108 cm^–1^ (C–N stretching) were observed (Figure S1). The PAAM PH beads were further used as substrates for the deposition of TiO_2_-, Al_2_O_3_-, and ZnO-nanocoatings by the alternating reactions of vaporized TiCl_4_, DEZ, or TMA and H_2_O with the amide functional groups on the surface of the voids. However, the 3D-interconnected porous morphology typical of the PH is crucial in these reactions as the gaseous precursors must diffuse readily through the structure to obtain precise control over the thickness of the deposited MO films (Scheme 1).

### Growth of TiO_2_-Nanocoatings in the PHs

**Figure 1:**
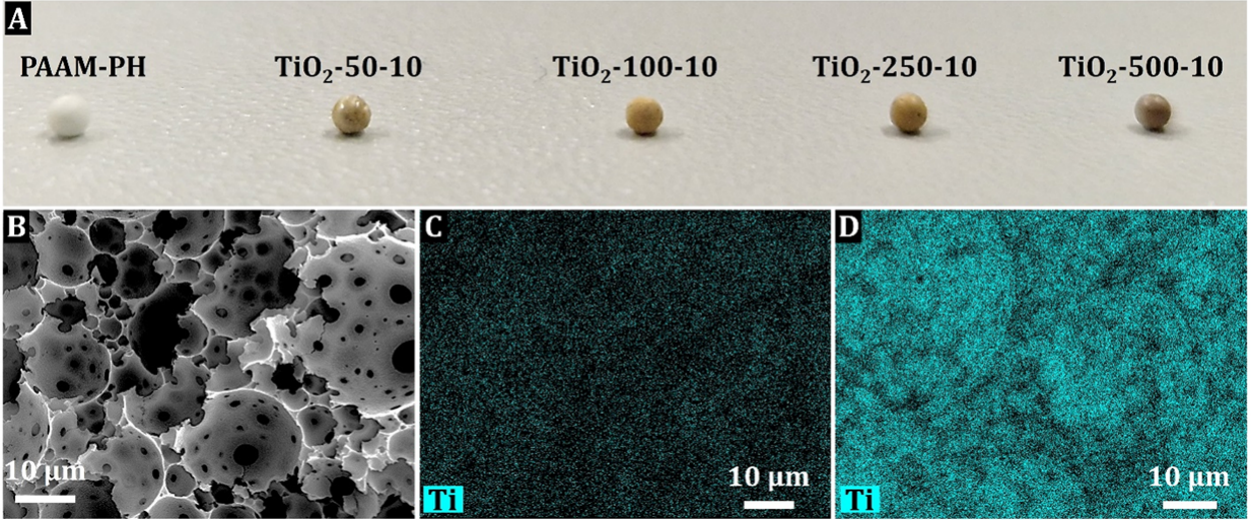
Photo of PAAM- and TiO_2_-coated PH beads (A); SEM micrographs of TiO_2_-500-10 PH (B); SEM-EDS elemental mapping for Ti atom of TiO_2_-50-10 (C); and TiO_2_-500-10 PH beads (D).

To understand how 3D-interconnected PH morphology affects the content, thickness, and growth per cycle (GPC) of TiO_2_ nanocoatings in the PAAM beads, TiCl_4_ was deposited with varying numbers of cycles. For this purpose, four sets of TiO_2_ PH samples were synthesized, i.e. TiO_2_-50-10, TiO_2_-100-10, TiO_2_-250-10, and TiO_2_-500-10 PH beads, differing in the number of coating cycles, namely 50, 100, 250, and 500 cycles at the same dosing time of 10 s per cycle. All resulting beads changed color from white to light brown after 50 ALD cycles to brown after 500 ALD cycles (Figures 1A and S2). The SEM analysis revealed a completely retained HIPE-templated macroporous structure after the TiO_2_ deposition, regardless of the cycle number, and the pore size also did not change compared to the net PAAM PH (Figure 1B).

This indicates sufficient thermal stability of the porous PAAM matrix to withstand the high temperatures during the ALD process (i.e., 250 °C in the case of TiCl_4_ deposition). The deposition of TiO_2_ inside the porous PH structure was further investigated by using SEM-EDS on cross-sectioned samples by tracking the Ti EDS mapping signal. The TiO_2_ content changes with the number of cycles, as seen in samples TiO_2_-50-10 and TiO_2_-500-10 (Figure 1C and D) and other samples in this series (Figure S3). Regardless of the amount of TiO_2_ deposited, TiCl_4_ precursor molecules were able to penetrate into the porous structure of PH in all cases, which allowed the facile formation of a TiO_2_ nanocoatings covering the voids’ surfaces in the PH beads. Moreover, the SEM-EDS line profile analysis data show a uniform distribution of Ti throughout the bead diameter (Figure S4), confirming that TiCl_4_ indeed penetrates the large PH structure covering the surface of the voids with atomic-control.

To quantify the amount of TiO_2_ as a function of coating cycles, we performed TGA measurements and determined the residual mass after heating the TiO_2_–PH nanocomposite beads in an air stream at 800 °C. First, the thermal behavior of pure PAAM PH was investigated, and it was found that the polymer network is stable up to about 270 °C and then completely decomposes when the temperature further increases to about 500 °C, as shown by the TGA analysis (Figure S5). Since the PAAM PH matrix already completely decomposes at 500 °C in the air stream, these residual masses, which were determined when TiO_2_–PH nanocomposites were heated at 800 °C, were directly related to TiO_2_. Thus, the total mass of TiO_2_ increases with the number of cycles from 2 wt % for TiO_2_-50 PH beads to 4, 7, and 19 wt % for TiO_2_-100, TiO_2_-250, and TiO_2_-500 PH beads, respectively (Figure S5).

**Figure 2:**
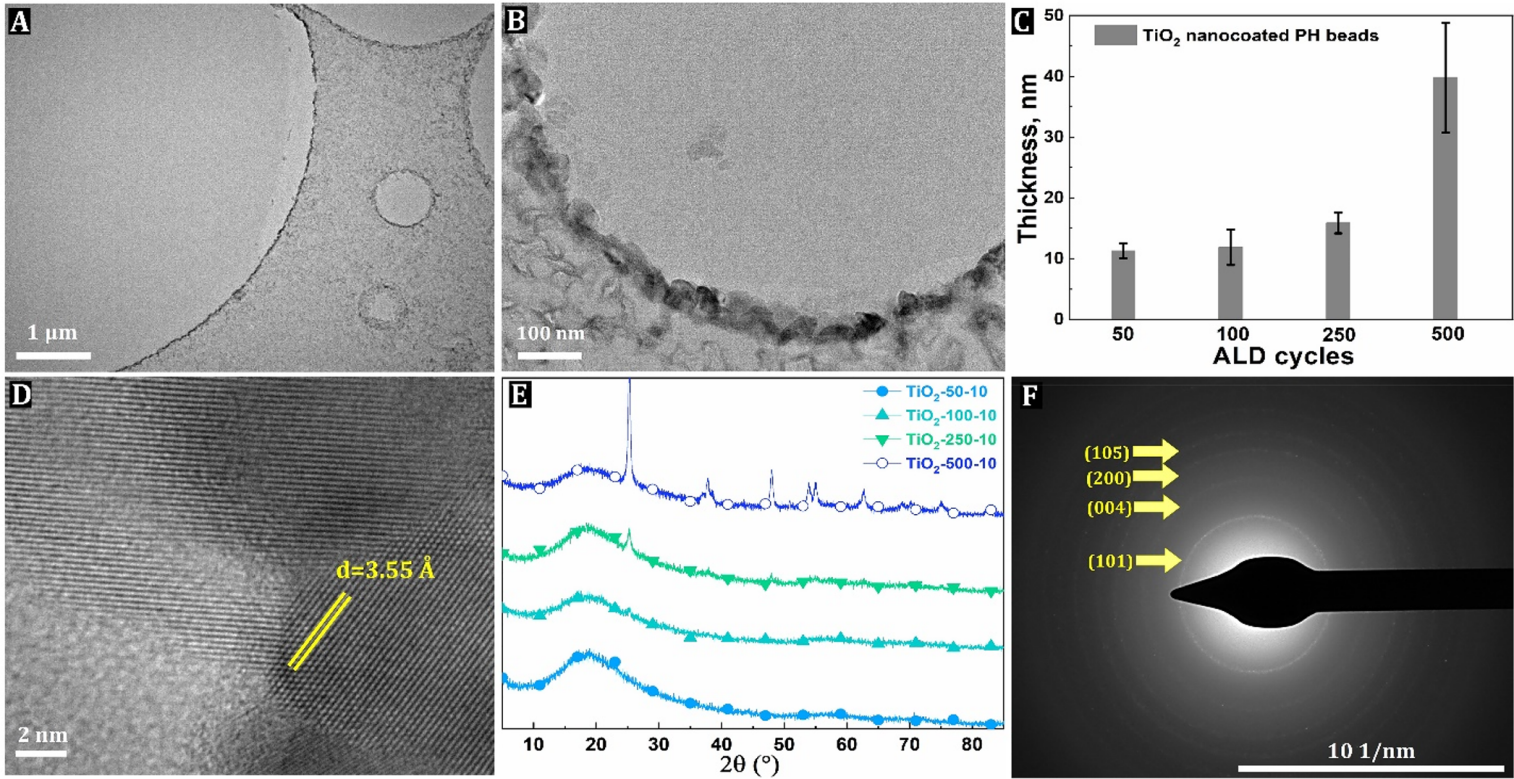
Elastic filtered TEM of TiO_2_-500-10 (A, B), thickness of TiO_2_ nanocoatings vs number of ALD cycles (C), high-resolution TEM of the TiO_2_-500-10 sample (D), XRD pattern of the TiO_2_-X-10 sample series (E), and selected-area electron diffraction (SAED) pattern of TiO_2_-500-10 (F).

TEM analysis was performed to further investigate the average thickness of the TiO_2_ nanocoatings within the PH structure in more detail. The TEM imaging of the nanocomposite beads showed that the ALD of TiO_2_ leads to the formation of a continuous coating over the surface of the void (Figure 2A, B). The thicknesses of the TiO_2_ coatings were about 11 ± 3, 11.9 ± 1, 16 ± 1, and 40 ± 9 nm for TiO_2_-50-10, TiO_2_-100-10, TiO_2_-250-10, and TiO_2_-500-10 PH beads, respectively (Figure 2C), corresponding to a GPC of about 0.24, 0.11, 0.07, and 0.08 nm.

The high-resolution TEM image shows that some TiO_2_ nanocoatings are also crystalline, exhibiting a large number of fringes (Figure 2D). Analysis of the fringe pattern of the TiO_2_-500-10 sample revealed an inter-reticular spacing of 0.355 nm, which is consistent with the (101) lattice plane of TiO_2_ anatase. The anatase structure is confirmed by X-ray diffraction (XRD) analysis with peaks at 2θ of 25.2°, 37.7°, 48.0°, 53.8°, 55.2°, and 62.7° corresponding to the (101), (004), (200), (105), (211), and (118) crystal planes of anatase (Figure 2E). The TiO_2_ crystal edge orientations were further analyzed using the corresponding electron diffraction spots in the SAED patterns, and the following edge orientations (101), (004), (200), and (105) were found in the TiO_2_-250-10 and TiO_2_-500-10 samples (Figures 2F and S6), but not in samples synthesized with fewer ALD cycles, i.e., TiO_2_-50-10 and TiO_2_-100-10 (Figure S6). The thickness and crystallization of TiO_2_ nanocoatings change with the number of ALD cycles, and this can be explained by the following hypotheses. Considering that the TiO_2_ film thickness increases slowly at the beginning of the ALD process, i.e., between 50 and 250 cycles, and a sharp increase in TiO_2_ film thickness is observed only at a higher cycle number, this indicates a so-called “delayed” growth mechanism (Figure 2C).^47^ The delayed-growth phenomenon is the result of the reactivity difference between the chemical sites on the polyacrylamide substrate containing amide functions and the as-grown TiO_2_ films containing hydroxyl functions. In the TiO_2_-50-10 and TiO_2_-100-10 samples, where we used 50 and 100 ALD cycles, respectively, we obtain thin TiO_2_ films because many amide functions are still present on the surface and they seem to be less reactive toward TiCl_4_/H_2_O precursor molecules. In contrast, thicker TiO_2_ films were obtained for the TiO_2_-250-10 and TiO_2_-500-10 samples, with a roughly 3-fold increase in thickness for TiO_2_-500-10 compared to the TiO_2_-50-10 sample. The rapid growth indicates a high density of available hydroxyl functions on the surface of the as-grown TiO_2_. Moreover, the film thickness was also found to be related to TiO_2_ crystallization, which is known as a thickness-dependent crystallization phenomenon.^48,49^ Literature reports that crystallization of TiO_2_ thin films is strongly affected by the dosing temperature in thermal ALD. TiO_2_ films deposited at dosing temperatures <165 °C are amorphous, while those deposited >165 °C should contain the anatase phase.^50^ Considering that all four TiO_2_-X-10 samples were in our case deposited at the same dosing temperature, i.e. 250 °C, it is intriguing that the thinner films in TiO_2_-50-10 and TiO_2_-100-10 developed as amorphous coatings, while the thicker ones in TiO_2_-250-10 and TiO_2_-500-10 exhibited crystalline properties. Crystallization thus appears to be thickness-dependent; i.e., it starts with individual crystal grains (S6A) that form after a specific number of ALD cycles, and above a certain TiO_2_ layer thickness, this crystallization process extends over the entire deposited film.^51,52^

While the number of ALD cycles obviously has an effect on the thickness and crystallization, we were also interested in the effect of TiCl_4_ dosing time on the formation of TiO_2_ nanocoatings. Therefore, three different dosing times were chosen, namely 30, 60, and 100 s at 50 ALD cycles program. The average thickness of the TiO_2_ nanocoatings was analyzed by TEM and found to be 16 ± 2, 4 ± 1, and 4 ± 0.5 nm after 30, 60, and 100 s dose time, respectively. Although no diffraction peaks belonging to anatase were found in XRD, we observed some weak diffraction spots in SAED patterns only in the TiO_2_-50-100 sample with the following edge orientations: (101), (200), (116), (103), and (211) (Figure S7). Finally, the residual masses were determined by TGA and found to be 4.5, 2.0, and 3.5 wt % for the TiO_2_-50-30, TiO_2_-50-60, and TiO_2_-50-100 PH beads, respectively (Figure S8). Apparently, not only the number of ALD cycles but also the dosing time can affect the thickness and crystalline nature of the TiO_2_ nanocoatings in the PAAM PH beads.

**Figure 3:**
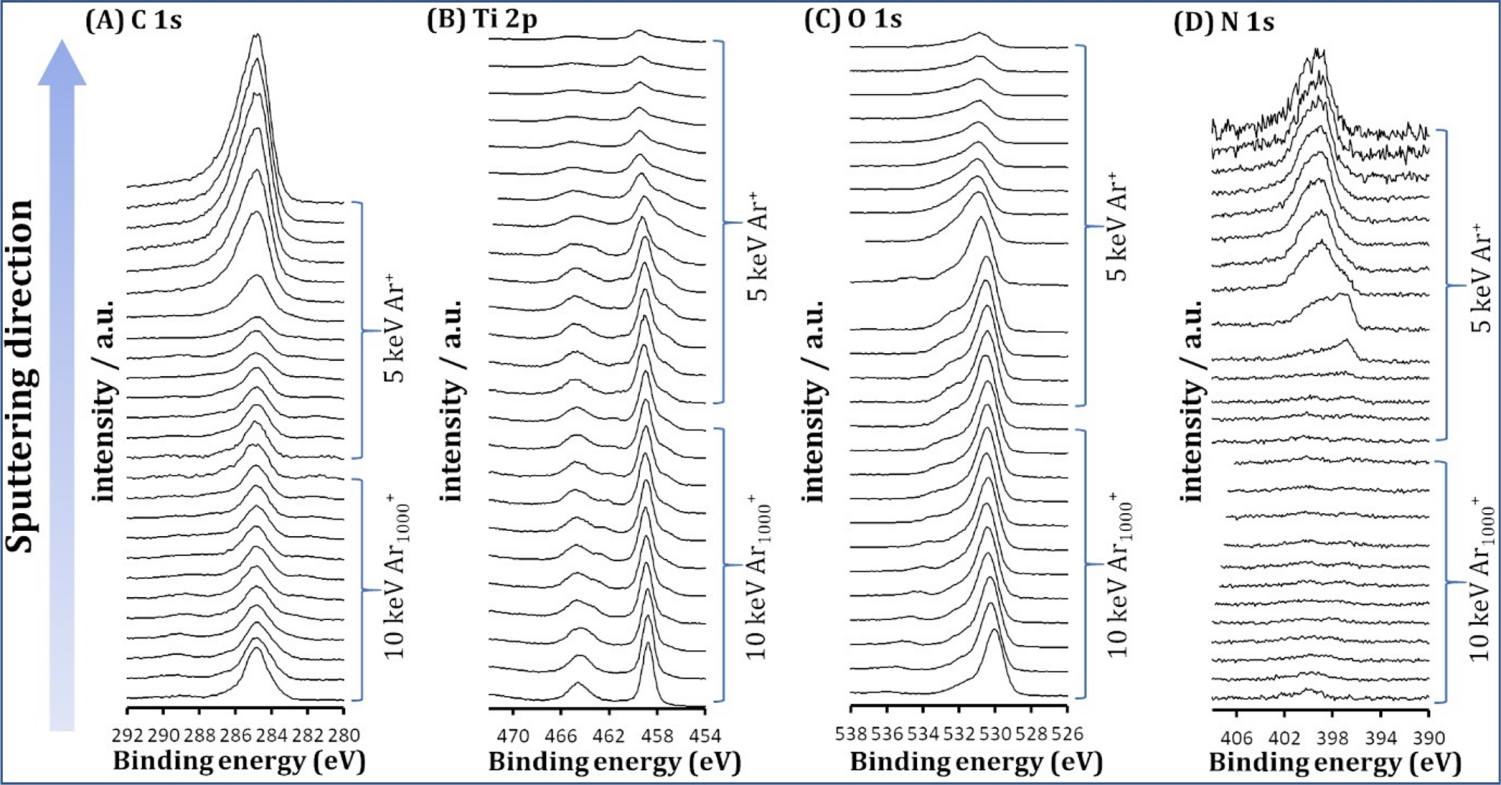
High-resolution XPS depth profiling of a TiO_2_-250-10 sample: C 1s (A), Ti 2p (B), O 1s (C), and N 1s (D).

XPS was used to analyze the surface composition of the voids in the TiO_2_-250-10 sample. The survey spectra showed the presence of Ti 2p, O 1s, N 1s, and C 1s signals (Figure S9), indicating the TiO_2_ and PAAM phases in the PH nanocomposite beads. To confirm the layered structure of the surface of the void and to determine whether TiO_2_ forms only at the surface or whether the precursor molecules (TiCl_4_/H_2_O) also penetrate and react in the underlying PAAM phase, depth profiling was performed by sputtering the surface associated with XPS analysis. Sputtering started by employing the Ar_1000_^+^ argon gas cluster ion beam (GCIB) at 10 keV followed by 5 keV monatomic Ar^+^ sputtering. High-resolution XPS spectra are shown in Figure 3. The surface of the voids in the uppermost position (the lowest spectra in Figure 3) shows the C-, Ti-, and O-containing species. The C atom probably comes from adventitious carbon contamination. The latter is supported by the fact that the main peak in the C 1s spectra loses intensity with sputtering the surface (Figure 3A), indicating the removal of adventitious carbonaceous species. The presence of the peak in the Ti 2p and the O 1s spectra is due to the TiO_2_ layer. Analysis of the high-resolution Ti 2p spectra revealed a doublet, i.e. Ti 2p_3/2_ and Ti 2p_1/2_ at binding energies of 458.7 and 464.4 eV, respectively, which corresponds to Ti^4+^/TiO_2_ (Figure 3B).^53^ The high-resolution XPS O 1s spectra before sputtering show the main peak at about 530.0 eV with a shoulder at a higher binding energy, indicating two different chemical environments of O-species. The main peak is attributed to O^2–^ in the TiO_2_ crystal structure, while the shoulder peak at higher bending energy is likely due to oxygen vacancies.^54^ Importantly, no N-containing species were found in the uppermost position (the lowest spectra in Figure 3D), suggesting a pure TiO_2_ layer (Figure 3D). After the tenth sputtering cycle, the main peak in the Ti 2p spectra began to decrease, indicating the proximity of the TiO_2_–PAAM interface. At this point, we continue with a 5 keV monoatomic Ar^+^. After the fifth sputtering cycle with 5 keV Ar^+^, a new peak appears in the N 1s spectrum at 396.8 eV, corresponding to TiO_2_–N bonding (Figure 3D),^55^ confirming the TiO_2_–PAAM interface. Upon further sputtering, the intensity of the peak in the N 1s spectra increases and shifts from 396.8 to 399.1 eV, which corresponds to the N atoms in the amide (Figure 3D).^56^ Moreover, the peak in the Ti 2p region eventually disappears, and the peak in the C 1s spectra gradually increases. Finally, a slight shift of the main peak in the O 1s region from 530.0 eV (O^2–^/TiO_2_) to 532.0 eV (O originating from C=O) (Figure 3C) confirms the PAAM phase. Thus, the XPS analysis corroborates that the TiO_2_ nanolayer occurred only on the void surface and that no TiO_2_ was found in the PAAM phase.

### ZnO and Al_2_O_3_ Nanocoated PHs

**Figure 4:**
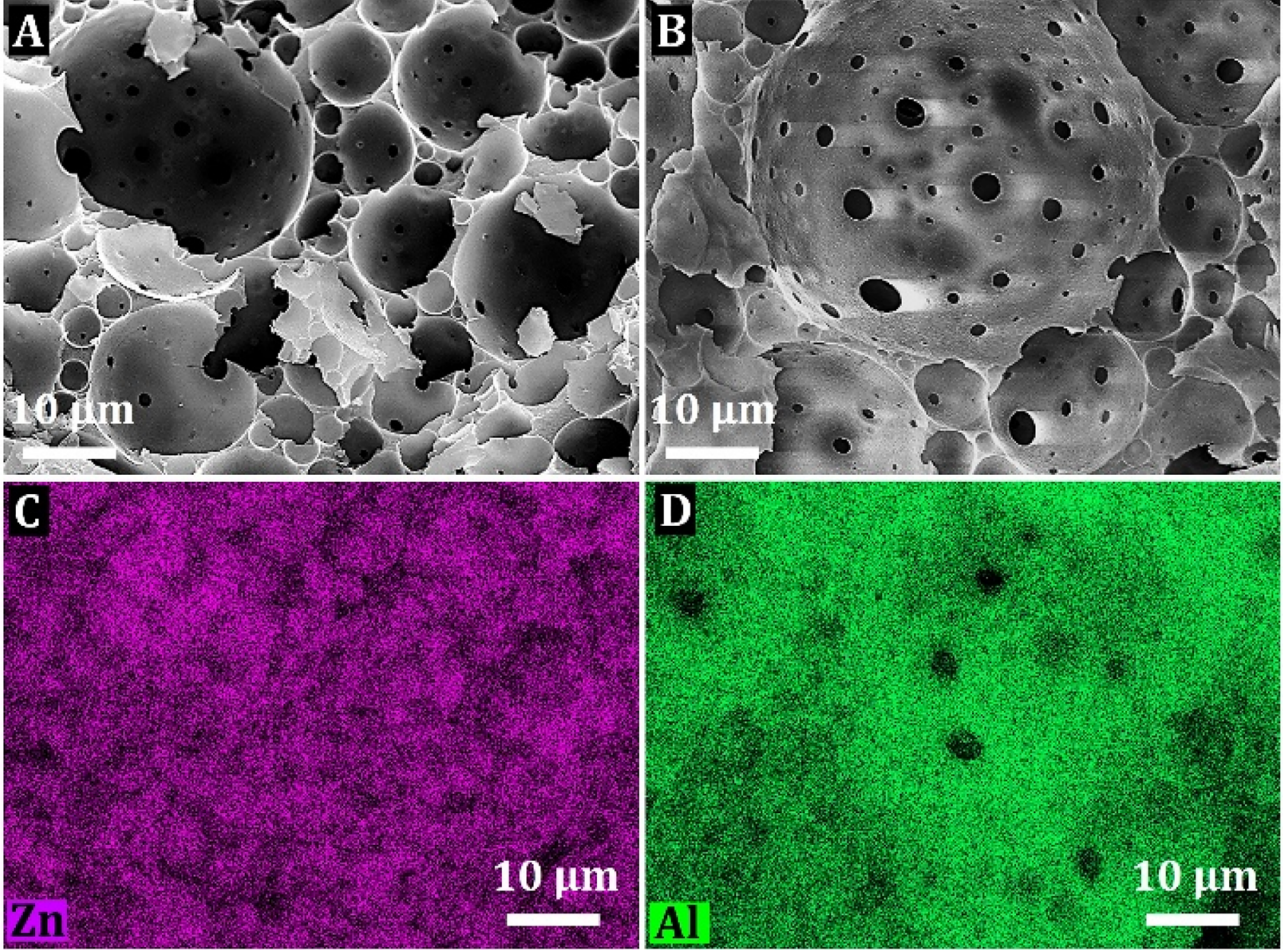
SEM images of ZnO-250-10 PH beads (A), Al_2_O_3_-250-10 PH beads (B), and SEM-EDS elemental mapping for the Zn atom of ZnO-250-10 PH beads (C) and Al atom of Al_2_O_3_-250-10 PH beads (D).

To demonstrate the versatility of the synthetic approach, we continued our studies and prepared thin atomic layer-deposited ZnO and Al_2_O_3_ nanocoatings on the inner surface of PH beads. Both metal oxide precursors, i.e., DEZ and TMA, were deposited with 250 ALD cycles and 10 s dosing time. The EDS analysis clearly shows the presence of Zn and Al atoms on the surface of the voids in the PH structure and thus the successful deposition of ZnO and Al_2_O_3_, respectively (Figure 4).

**Figure 5:**
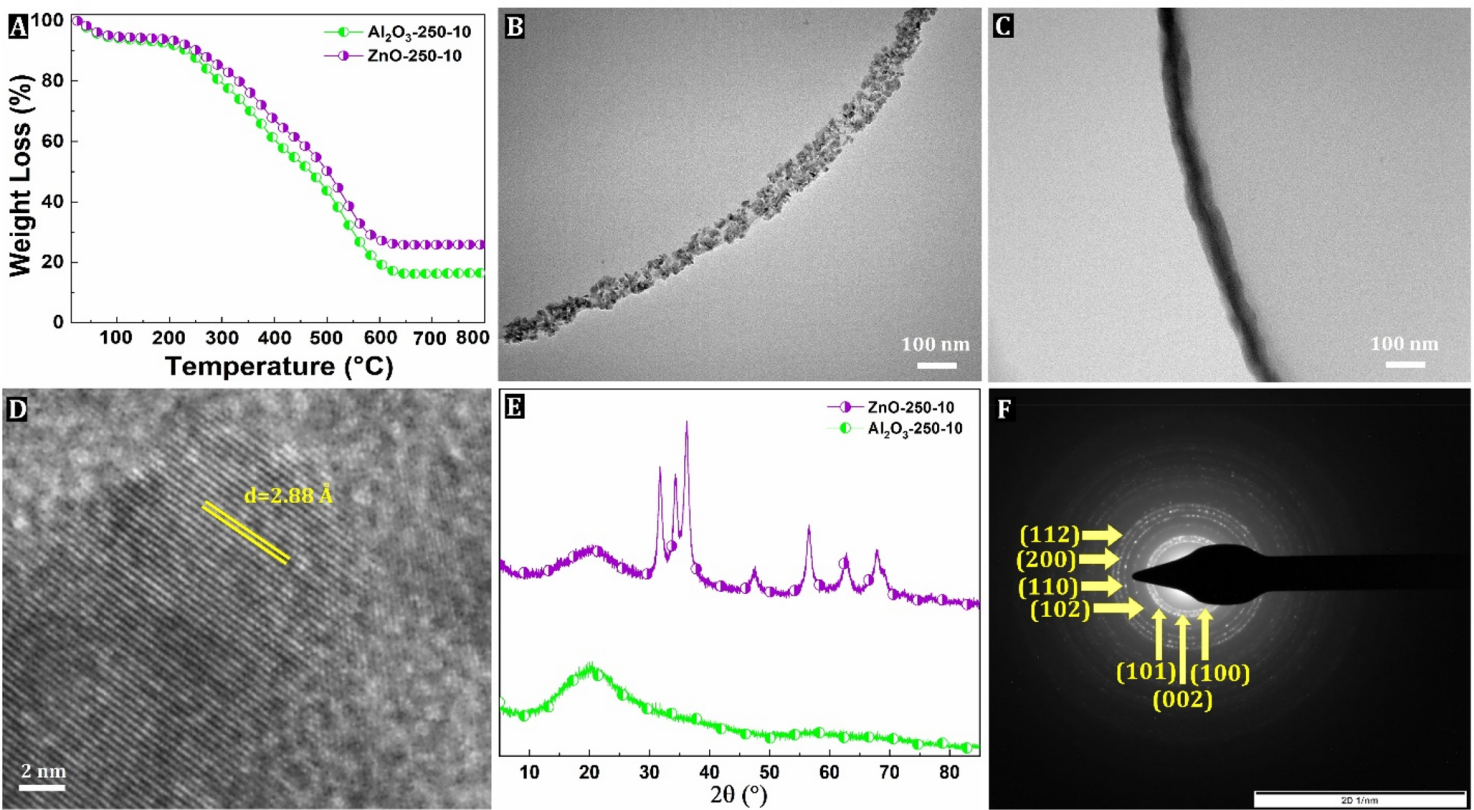
TGA of ZnO- and Al_2_O_3_-250-10 sample (A), elastic filtered TEM of ZnO- and Al_2_O_3_-250-10 sample (B, C), high-resolution TEM of the ZnO-250-10 sample (D), XRD pattern of the ZnO- and Al_2_O_3_-250-10 sample (E), and selected-area electron diffraction (SAED) pattern of ZnO-250-10 sample (F).

Thermogravimetric analysis indeed confirmed the presence of ZnO and Al_2_O_3_ in the PAAM matrix. The residual masses were 26 and 16 wt % for the ZnO-250-10 and Al_2_O_3_-250-10 samples, respectively (Figure 5A). The average thickness of the ZnO and Al_2_O_3_ nanocoatings determined by TEM imaging was found to be about 31 ± 6 and 74 ± 28 nm, respectively (Figure 5B and C), corresponding to a GPC of about 0.12 and 0.29 nm under these experimental conditions. Interestingly, the GPC values for ZnO-250-10 and Al_2_O_3_-250-10 are both higher than that of TiO_2_-250-10 PH, although the opposite would be expected. Indeed, steric hindrance from chemisorbed neighboring precursor molecules at the polymer surface is lower when smaller TiCl_4_ molecules are deposited than for larger DEZ and TMA molecules, which affects the concentration of the accessible reactive surface sites. However, the main reason for the lower growth rate of TiO_2_ is probably secondary reactions, namely the chlorination of the substrate surface by HCl formed during the TiCl_4_/H_2_O process, which temporarily blocks the adsorption sites for TiCl_4_ and reduces the growth rate and thickness of TiO_2_ nanocoatings.^57,58^

The high-resolution TEM analysis shows that ZnO nanocoatings are also crystalline, exhibiting a large number of fringes (Figure 5D). Analysis of the fringe pattern of the ZnO-500-10 sample revealed an inter-reticular spacing of 0.288 nm, consistent with the (100) lattice plane of ZnO wurtzite. The XRD diffraction patterns are shown in Figure 5E. In the case of ZnO-250-10, typical diffraction peaks at 2θ of 31.5°, 34.6°, 36.1°, 47.2°, 57.1°, 62.8°, and 67.9° correspond to crystal planes (100), (002), (101), (102), (110), (103), and (112) of hexagonal wurtzite ZnO. Most of these planes were also corroborated by corresponding electron diffraction spots in the SAED pattern, and the following ZnO crystal edge orientations found at (200), (002), (101), (102), (110), (100), and (112) (Figure 5F). On the other hand, the XRD diffraction pattern of the Al_2_O_3_-250-10 sample revealed an amorphous structure without diffraction peaks as well as without detectable electron diffraction spots in the SAED (Figure S10).^59^

The XPS survey spectra measurements for Al_2_O_3_- and ZnO-250-10 samples showed the presence of C 1s, N 1s, and O 1s signals along with Zn 2p, Zn 3s, and Zn 3p XPS signals and XPS-induced Auger LMM signals for ZnO, and Al 2s and 2p signals for Al_2_O_3_, clearly indicating the presence of ZnO and Al_2_O_3_ at the surface of the voids in PAAM PH beads (Figure S11). Moreover, high-resolution XPS spectral measurements were performed for ZnO, Al_2_O_3_, and PAAM and are shown in Figure S12. The peak in the C 1s spectrum at a binding energy of 285.6 eV can be assigned to C–N in the amide group of PAAM (Figure S12A). In addition, the C 1s spectrum in ZnO-250-10 reveals a second peak at 289.15 eV, which is probably related to the COO groups that appear after amide hydrolysis in PAAM.^60^ COO is also seen in the spectral O 1s spectrum at 534.23 eV. Interestingly, this peak is not seen in the other two samples, i.e., TiO_2_- and Al_2_O_3_-250-10. Hydrolysis of the amide group leading to a carboxylic acid group within the PAAM macromolecular backbone can indeed occur by different mechanistic pathways.^61^ In our case, therefore, amide hydrolysis is likely related to the different conditions under which precursor molecules react with surface functional groups to form MO. The presence of PAAM can be further confirmed by the high-resolution XPS spectra of the N 1s and the O 1s orbitals exhibiting peaks at 399.7 and 532.0 eV, respectively (Figure S12B,C). The Al 2p region revealed a single peak at 74.1 eV corresponding to Al^3+^/Al_2_O_3_ (Figure S12D), while the Zn 2p spectrum showed a doublet at 1021.5 and 1044.6 eV, which corresponds to the Zn 2p_3/2_ and Zn 2p_1/2_, respectively, in the Zn^2+^/ZnO (Figure S12E).^62,63^

### Photocatalytic Oxidation of Bisphenol A (BPA)

**Figure 6:**
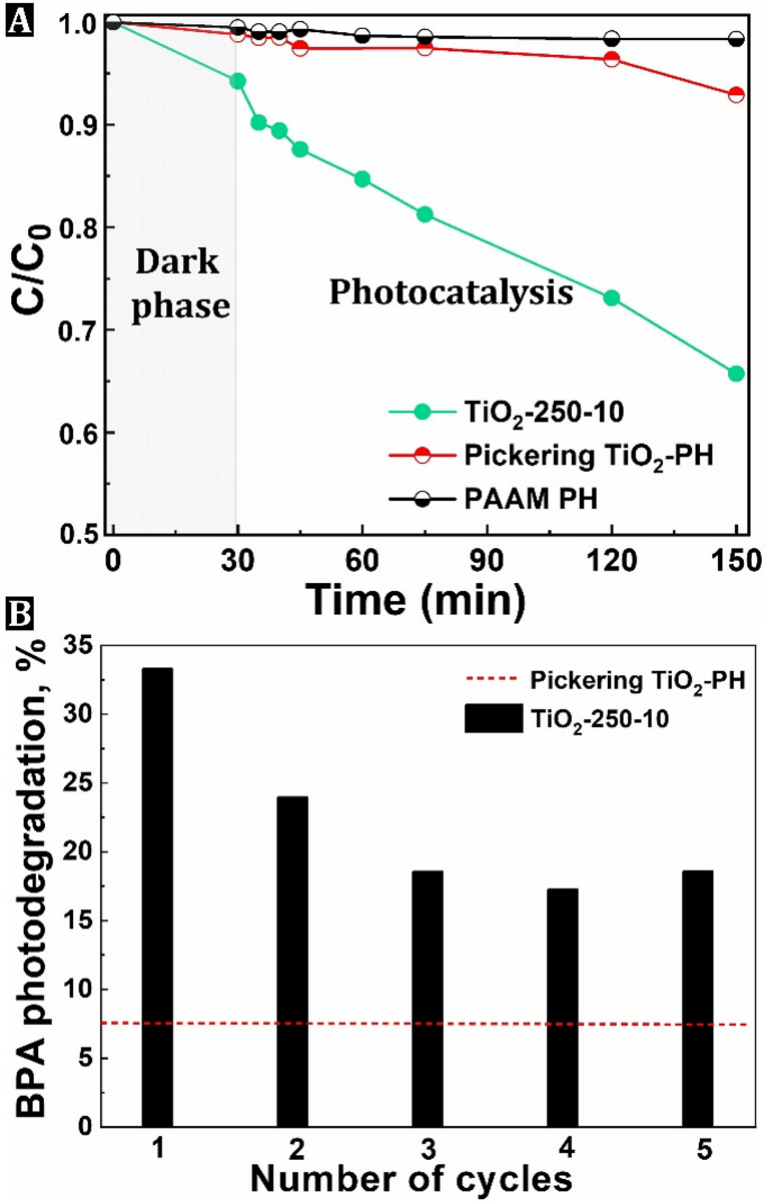
Photocatalytic oxidation of an aqueous solution of BPA carried out in the presence of PH catalysts irradiated with UV light (A) and BPA photodegradation performance during 5 cycles using the same catalyst batch (B).

The photocatalytic activity of the developed TiO_2_-250-10 beads as heterogeneous catalysts was determined for the oxidative photodegradation of BPA dissolved in water and compared with that of TiO_2_–PH beads prepared by the conventional Pickering method. It should be noted that the PH beads obtained by both routes contained the same amount of TiO_2_ in the structure, i.e., ∼10 wt %, and differed only by spatial distribution. The SEM-EDS elemental mapping for Ti in the Pickering beads’ PH revealed that TiO_2_ is mainly present in the PAAM phase and less at the surface (Figure S13). Namely, we and others have shown that in the Pickering PHs, MOs are present both on the surface of the voids and in the bulk of the PH skeleton.^18,64,65^ However, as we show in this paper, ALD-derived PH nanocomposites have MO exclusively on the surface of the voids, which should be beneficial for applications such as heterogeneous catalysis and advantageous over the Pickering PH nanocomposites that we will show as an example. Figure 6A shows a comparison of photocatalytic activity exerted by various samples. Control experiments conducted with PAAM beads without TiO_2_ or without UV light illumination showed no BPA degradation. The ALD-derived TiO_2_–PH beads showed rapid photocatalytic degradation of BPA in an aqueous solution and could photodegrade about 30% BPA within 120 min. In contrast, the Pickering PH beads reacted very slowly after the light was turned on and exhibited some photocatalytic activity within 120 min, i.e. ∼5%. Interestingly, in the “dark phase” (experimental phase without light), about 6% of the BPA is adsorbed in the ALD-derived TiO_2_ PH beads but not in the net PAAM, which means that this must be related to the TiO_2_ layer on the surface and not to the highly porous PH structure. However, the adsorption apparently has no effect on the further photocatalytic efficiency since the ALD TiO_2_–PH photocatalyst still achieves a BPA degradation of 30% within 120 min without reaching the plateau. Moreover, ALD derived TiO_2_–PH beads can be easily recovered and reused. After five consecutive runs, some diminution in the photocatalytic performance is observed. It decreased by 15% between the first and third runs and remained at a similar level until the fifth run (Figure 6B). The decrease in BPA degradation could be due to accumulation and deposition of the photochemical degradation products on the surface of the TiO_2_ nanocoating. Although the photocatalytic activity of the TiO_2_-250-10 sample decreases after five runs using the same batch of catalyst, it is still much better than the performance of the Pickering TiO_2_-PH beads in the first cycle (Figure 6B). Finally, to confirm the robustness of the synthesized TiO_2_ layer on the PH surface, XPS analysis of the beads was performed after the fifth photocatalytic cycle. The XPS spectrum of the TiO_2_-250-10 sample is shown in Figure S14 and reveals the Ti 2p, Ti 3s, Ti 3p, O 1s, and C 1s signals corresponding to TiO_2_. This confirms the presence of TiO_2_ in the PH nanocomposite beads, even after five consecutive photocatalytic runs in the aqueous medium. The results of the experiments on photocatalytic oxidation of an aqueous BPA solution (Figure 6) show that the efficiency of the ALD TiO_2_–PH sample is significantly higher than the efficiency of the Pickering TiO_2_–PH sample, which we attribute to the more suitable deposition of the TiO_2_ phase and thus easier access of the BPA molecules dissolved in water to the active sites as well as more efficient use of UV light. The ALD method of depositing the TiO_2_ phase, compared to conventional methods of material preparation, may also have an impact on the optical and electronic properties of the photocatalysts and, consequently, on the efficiency of formation of reactive oxygen species involved in the photocatalytic oxidation process. The latter remains the subject of further research.

## 4 Conclusions

In summary, atomic-layer deposition has been successfully used for 3D surface nanofunctionalization of macroporous PH beads. A key advantage of this technique is the controlled atomic-scale thickness of the deposited layers on the surface of the voids in the PH structure. The successful synthesis of the MO-PH beads thus obtained was confirmed by TGA, SEM, EDS, XRD, TEM, and XPS analyses. The combination of these characterization techniques showed that both the number of ALD cycles and the dosing time have an influence on the total mass, thickness, and crystallization of the obtained MO layers in the PAAM PH. TGA showed between 2 and 19 wt % TiO_2_ and about 26 and 16 wt % ZnO and Al_2_O_3_, respectively. The primary macroporous morphology of PHs was not affected by the ALD process, as shown by SEM, although in some cases the vaporization temperature reached 250 °C. The results of the EDS examination indicated that MOs were deposited on the entire inner and outer surfaces of PAAM PH beads. TEM analysis revealed nanolayers on the inner highly porous PH surfaces with thicknesses between 11.3 ± 3 and 40 ± 9 nm for TiO_2_ and 31 ± 6 and 74 ± 28 nm for ZnO and Al_2_O_3_, respectively. Further combination of TEM and XRD measurements confirmed a thickness-dependent crystallization behavior, with TiO_2_ nanolayers appearing as an amorphous structure at 50 or 100 cycles and beginning to crystallize at 250 cycles of ALD. In the case of Al_2_O_3_, we could not detect any crystalline structure under these ALD conditions. Qualitative XPS analysis enabled the confirmation of the chemical composition of the respective nanocoatings by identifying specific species, namely, Ti^4+^/TiO_2_, Zn^2+^/ZnO, and Al^3+^/Al_2_O_3_. Moreover, the XPS depth profiling of the TiO_2_–PH sample displayed a TiO_2_ nanolayer formed exclusively on the surface of the voids and confirmed that the precursor molecules did not penetrate into the underlying PAAM phase. Finally, the advantage of this surface coverage by TiO_2_ in the ALD-derived TiO_2_ PH beads was demonstrated through the photocatalytic oxidation of BPA. It has been shown that the efficiency is significantly better compared to Pickering TiO_2_ PH beads.

The combination of atomic-layer deposition and emulsion-templated macroporous structures opens up the possibility of creating PH nanocomposites with well-defined and easily accessible MO layers. However, it is important to note that the functionality is not limited only to the combination of TiO_2_, ZnO, or Al_2_O_3_ in PAAM PH shown in this work but can be considered as a more universal method for functionalization of PH that can also include biological materials such as peptides or enzymes.

## Supporting Information Available

Details of material synthesis, and characterization supplementary figures (Figures S1–S12), and supplementary table (Table S1) (PDF)

## References

[ref1] Wu D.; Xu F.; Sun B.; Fu R.; He H.; Matyjaszewski K. Design and Preparation of Porous Polymers. Chem. Rev. 2012, 112 (7), 3959–4015. 10.1021/cr200440z. 22594539

[ref2] Poupart R.; Grande D.; Carbonnier B.; Le Droumaguet B. Porous Polymers and Metallic Nanoparticles: A Hybrid Wedding as a Robust Method toward Efficient Supported Catalytic Systems. Prog. Polym. Sci. 2019, 96, 21–42. 10.1016/j.progpolymsci.2019.05.003.

[ref3] Chen L.; Rende D.; Schadler L. S.; Ozisik R. Polymer Nanocomposite Foams. J. Mater. Chem. A 2013, 1, 3837–3850. 10.1039/c2ta00086e.

[ref4] Peponi L.; Puglia D.; Torre L.; Valentini L.; Kenny J. M. Processing of Nanostructured Polymers and Advanced Polymeric Based Nanocomposites. Mater. Sci. Eng. R Rep. 2014, 85, 1–46. 10.1016/j.mser.2014.08.002.

[ref5] Moon S.; Kim J. Q.; Kim B. Q.; Chae J.; Choi S. Q. Processable Composites with Extreme Material Capacities: Toward Designer High Internal Phase Emulsions and Foams. Chem. Mater. 2020, 32 (11), 4838–4854. 10.1021/acs.chemmater.9b04952.

[ref6] Cameron N. R.; Sherrington D. C. High Internal Phase Emulsions (HIPEs) - Structure, Properties and Use in Polymer Preparation. Adv. Polym. Sci. 1996, 126, 163–214. 10.1007/3-540-60484-7_4.

[ref7] Stubenrauch C.; Menner A.; Bismarck A.; Drenckhan W. Emulsion and Foam Templating-Promising Routes to Tailor-Made Porous Polymers. Angew. Chem. - Int. Ed. 2018, 57 (32), 10024–10032. 10.1002/anie.201801466. 29701918

[ref8] Foudazi R. HIPEs to PolyHIPEs. React. Funct. Polym. 2021, 164, 104917 10.1016/j.reactfunctpolym.2021.104917.

[ref9] Brun N.; Ungureanu S.; Deleuze H.; Backov R. Hybrid Foams, Colloids and beyond: From Design to Applications. Chem. Soc. Rev. 2011, 40 (2), 771–788. 10.1039/B920518G. 21088777

[ref10] Silverstein M. S. PolyHIPEs: Recent Advances in Emulsion-Templated Porous Polymers. Prog. Polym. Sci. 2014, 39 (1), 199–234. 10.1016/j.progpolymsci.2013.07.003.

[ref11] Haibach K.; Menner A.; Powell R.; Bismarck A. Tailoring Mechanical Properties of Highly Porous Polymer Foams: Silica Particle Reinforced Polymer Foams via Emulsion Templating. Polymer (Guildf) 2006, 47 (13), 4513–4519. 10.1016/j.polymer.2006.03.114.

[ref12] Wu R.; Menner A.; Bismarck A. Tough Interconnected Polymerized Medium and High Internal Phase Emulsions Reinforced by Silica Particles. J. Polym. Sci., Part A: Polym. Chem. 2010, 48 (9), 1979–1989. 10.1002/pola.23965.

[ref13] Silverstein M. S. Emulsion-Templated Porous Polymers: A Retrospective Perspective. Polymer 2014, 55, 304–320. 10.1016/j.polymer.2013.08.068.

[ref14] Menner A.; Verdejo R.; Shaffer M.; Bismarck A. Particle-Stabilized Surfactant-Free Medium Internal Phase Emulsions as Templates for Porous Nanocomposite Materials: Poly-Pickering-Foams. Langmuir 2007, 23 (5), 2398–2403. 10.1021/la062712u. 17309201

[ref15] Cohen N.; Samoocha D. C.; David D.; Silverstein M. S. Carbon Nanotubes in Emulsion-Templated Porous Polymers: Polymer Nanoparticles, Sulfonation, and Conductivity. J. Polym. Sci., Part A: Polym. Chem. 2013, 51 (20), 4369–4377. 10.1002/pola.26851.

[ref16] Wong L. L. C.; Barg S.; Menner A.; Do Vale Pereira P.; Eda G.; Chowalla M.; Saiz E.; Bismarck A. Macroporous Polymer Nanocomposites Synthesised from High Internal Phase Emulsion Templates Stabilised by Reduced Graphene Oxide. Polymer (Guildf) 2014, 55 (1), 395–402. 10.1016/j.polymer.2013.09.039.

[ref17] Brown E. E. B.; Woltornist S. J.; Adamson D. H. PolyHIPE Foams from Pristine Graphene: Strong, Porous, and Electrically Conductive Materials Templated by a 2D Surfactant. J. Colloid Interface Sci. 2020, 580, 700–708. 10.1016/j.jcis.2020.07.026. 32712476

[ref18] Kovačič S.; Matsko N. B.; Ferk G.; Slugovc C. Macroporous Poly(Dicyclopentadiene) γFe_2_O _3_/Fe_3_O_4_ Nanocomposite Foams by High Internal Phase Emulsion Templating. J. Mater. Chem. A 2013, 1 (27), 7971–7978. 10.1039/c3ta11402c.

[ref19] Mudassir M. A.; Hussain S. Z.; Jilani A.; Zhang H.; Ansari T. M.; Hussain I. Magnetic Hierarchically Macroporous Emulsion-Templated Poly(Acrylic Acid)-Iron Oxide Nanocomposite Beads for Water Remediation. Langmuir 2019, 35 (27), 8996–9003. 10.1021/acs.langmuir.9b01121. 31189312

[ref20] Recio-Colmenares C. L.; Ortíz-Rios D.; Pelayo-Vázquez J. B.; Moreno-Medrano E. D.; Arratia-Quijada J.; Torres-Lubian J. R.; Huerta-Marcial S. T.; Mota-Morales J. D.; Pérez-García M. G. Polystyrene Macroporous Magnetic Nanocomposites Synthesized through Deep Eutectic Solvent-in-Oil High Internal Phase Emulsions and Fe_3_O_4_ Nanoparticles for Oil Sorption. ACS Omega 2022, 7 (25), 21763–21774. 10.1021/acsomega.2c01836. 35785308 PMC9245104

[ref21] Zhang T.; Cao H.; Gui H.; Xu Z.; Zhao Y. Microphase-Separated, Magnetic Macroporous Polymers with Amphiphilic Swelling from Emulsion Templating. Polym. Chem. 2022, 13 (8), 1090–1097. 10.1039/D1PY01584B.

[ref22] Rizvi A.; Chu R. K. M.; Park C. B. Scalable Fabrication of Thermally Insulating Mechanically Resilient Hierarchically Porous Polymer Foams. ACS Appl. Mater. Interfaces 2018, 10 (44), 38410–38417. 10.1021/acsami.8b11375. 30360118

[ref23] Horvat G.; Kotnik T.; Žvab K.; Knez Ž.; Novak Z.; Kovačič S. Silica Aerogel-Filled Polymer Foams by Emulsion-Templating: One-Pot Synthesis, Hierarchical Architecture and Thermal Conductivity. Chem. Eng. J. 2022, 450, 138251 10.1016/j.cej.2022.138251.

[ref24] Ye Y.; Jin M.; Wan D. One-Pot Synthesis of Porous Monolith-Supported Gold Nanoparticles as an Effective Recyclable Catalyst. J. Mater. Chem. A 2015, 3 (25), 13519–13525. 10.1039/C5TA02925B.

[ref25] Kovačič S.; Mazaj M.; Ješelnik M.; Pahovnik D.; Žagar E.; Slugovc C.; Logar N. Z. Synthesis and Catalytic Performance of Hierarchically Porous MIL-100(Fe)@polyHIPE Hybrid Membranes. Macromol. Rapid Commun. 2015, 36 (17), 1605–1611. 10.1002/marc.201500241. 26173197

[ref26] Yeşil R.; Çetinkaya S. Mn_3_O_4_/p(DCPD)HIPE Nanocomposites as an Efficient Catalyst for Oxidative Degradation of Phenol. J. Nanopart. Res. 2020, 22 (7), 1–14. 10.1007/s11051-020-04931-6.35517915

[ref27] Kim D.; Kim H.; Chang J. Y. Designing Internal Hierarchical Porous Networks in Polymer Monoliths That Exhibit Rapid Removal and Photocatalytic Degradation of Aromatic Pollutants. Small 2020, 16 (22), 1907555 10.1002/smll.201907555. 32348034

[ref28] Mazaj M.; Logar N. Z.; Žagar E.; Kovačič S. A Facile Strategy towards a Highly Accessible and Hydrostable MOF-Phase within Hybrid PolyHIPEs through in Situ Metal-Oxide Recrystallization. J. Mater. Chem. A 2017, 5 (5), 1967–1971. 10.1039/C6TA10886E.

[ref29] Zhu J.; Wu L.; Bu Z.; Jie S.; Li B. G. Polyethylenimine-Grafted HKUST-Type MOF/PolyHIPE Porous Composites (PEI@PGD-H) as Highly Efficient CO_2_ Adsorbents. Ind. Eng. Chem. Res. 2019, 58 (10), 4257–4266. 10.1021/acs.iecr.9b00213.

[ref30] Mazaj M.; Bjelica M.; Žagar E.; Logar N. Z.; Kovačič S. Zeolite Nanocrystals Embedded in Microcellular Carbon Foam as a High-Performance CO_2_ Capture Adsorbent with Energy-Saving Regeneration Properties. ChemSusChem 2020, 13 (8), 2089–2097. 10.1002/cssc.201903116. 31968150

[ref31] Vrtovec N.; Jurjevec S.; Zabukovec Logar N.; Mazaj M.; Kovačič S. Metal Oxide-Derived MOF-74 Polymer Composites through Pickering Emulsion-Templating: Interfacial Recrystallization, Hierarchical Architectures, and CO_2_ Capture Performances. ACS Appl. Mater. Interfaces 2023, 15 (14), 18354–18361. 10.1021/acsami.3c01796. 36996820 PMC10103051

[ref32] Desforges A.; Backov R.; Deleuze H.; Mondain-Monval O. Generation of Palladium Nanoparticles within Macrocellular Polymeric Supports: Application to Heterogeneous Catalysis of the Suzuki-Miyaura Coupling Reaction. Adv. Funct. Mater. 2005, 15 (10), 1689–1695. 10.1002/adfm.200500146.

[ref33] Zhang H.; Hussain I.; Brust M.; Cooper A. I. Emulsion-Templated Gold Beads Using Gold Nanoparticles as Building Blocks. Adv. Mater. 2004, 16 (1), 27–30. 10.1002/adma.200306153.

[ref34] Zhang H.; Hardy G. C.; Khimyak Y. Z.; Rosseinsky M. J.; Cooper A. I. Synthesis of Hierarchically Porous Silica and Metal Oxide Beads Using Emulsion-Templated Polymer Scaffolds. Chem. Mater. 2004, 16 (22), 4245–4256. 10.1021/cm0492944.

[ref35] Zhang T.; Sanguramath R. A.; Israel S.; Silverstein M. S. Emulsion Templating: Porous Polymers and Beyond. Macromolecules 2019, 52 (15), 5445–5479. 10.1021/acs.macromol.8b02576.

[ref36] Johnson R. W.; Hultqvist A.; Bent S. F. A Brief Review of Atomic Layer Deposition: From Fundamentals to Applications. Mater. Today 2014, 17, 236–246. 10.1016/j.mattod.2014.04.026.

[ref37] Keuter T.; Menzler N. H.; Mauer G.; Vondahlen F.; Vaßen R.; Buchkremer H. P. Modeling Precursor Diffusion and Reaction of Atomic Layer Deposition in Porous Structures. J. Vac. Sci. Technol. A 2015, 33 (1), 01A104 10.1116/1.4892385.

[ref38] Detavernier C.; Dendooven J.; Pulinthanathu Sree S.; Ludwig K. F.; Martens J. A. Tailoring Nanoporous Materials by Atomic Layer Deposition. Chem. Soc. Rev. 2011, 40 (11), 5242–5253. 10.1039/c1cs15091j. 21695333

[ref39] George S. M. Atomic Layer Deposition: An Overview. Chem. Rev. 2010, 110 (1), 111–131. 10.1021/cr900056b. 19947596

[ref40] Wu L.; Shi S.; Wang G.; Mou P.; Liu X.; Liu J.; Li L.; Du C. Carbon Nanocoils/Carbon Foam as the Dynamically Frequency-Tunable Microwave Absorbers with an Ultrawide Tuning Range and Absorption Bandwidth. Adv. Funct. Mater. 2022, 32 (52), 2209898 10.1002/adfm.202209898.

[ref41] Xu X.; Shi S.; Tang Y.; Wang G.; Zhou M.; Zhao G.; Zhou X.; Lin S.; Meng F. Growth of NiAl-Layered Double Hydroxide on Graphene toward Excellent Anticorrosive Microwave Absorption Application. Adv. Sci. 2021, 8 (5), 2002658 10.1002/advs.202002658. PMC7927622 33717840

[ref42] Kircher L.; Theato P.; Cameron N. R. Functionalization of Porous Polymers from High-Internal-Phase Emulsions and Their Applications. In Functional Polymers by Post-Polymerization Modification; Wiley: 2012; pp 333–352.

[ref43] Jurjevec S.; Debuigne A.; Žagar E.; Kovačič S. An Environmentally Benign Post-Polymerization Functionalization Strategy towards Unprecedented Poly(Vinylamine) PolyHIPEs. Polym. Chem. 2021, 12 (8), 1155–1164. 10.1039/D0PY01677B.

[ref44] Liang X.; Lynn A. D.; King D. M.; Bryant S. J.; Weimer A. W. Biocompatible Interface Films Deposited within Porous Polymers by Atomic Layer Deposition (ALD). ACS Appl. Mater. Interfaces 2009, 1 (9), 1988–1995. 10.1021/am9003667. 20355824

[ref45] Liang X.; George S. M.; Weimer A. W.; Li N. H.; Blackson J. H.; Harris J. D.; Li P. Synthesis of a Novel Porous Polymer/Ceramic Composite Material by Low-Temperature Atomic Layer Deposition. Chem. Mater. 2007, 19 (22), 5388–5394. 10.1021/cm071431k.

[ref46] Jurjevec S.; Žagar E.; Pahovnik D.; Kovačič S. Highly Porous Polyelectrolyte Beads through Multiple-Emulsion-Templating: Synthesis and Organic Solvent Drying Efficiency. Polymer (Guildf) 2021, 212, 123166 10.1016/j.polymer.2020.123166.

[ref47] Puurunen R. L.; Vandervorst W. Island Growth as a Growth Mode in Atomic Layer Deposition: A Phenomenological Model. J. Appl. Phys. 2004, 96 (12), 7686–7695. 10.1063/1.1810193.

[ref48] Cho C. J.; Kang J. Y.; Lee W. C.; Baek S. H.; Kim J. S.; Hwang C. S.; Kim S. K. Interface Engineering for Extremely Large Grains in Explosively Crystallized TiO_2_ Films Grown by Low-Temperature Atomic Layer Deposition. Chem. Mater. 2017, 29 (5), 2046–2054. 10.1021/acs.chemmater.6b04090.

[ref49] Chung H. K.; Won S. O.; Park Y.; Kim J. S.; Park T. J.; Kim S. K. Atomic-Layer Deposition of TiO2 Thin Films with a Thermally Stable (CpMe5)Ti(OMe)3 Precursor. Appl. Surf. Sci. 2021, 550, 149381 10.1016/j.apsusc.2021.149381.

[ref50] Aarik J.; Aidla A.; Uustare T.; Sammelselg V. Morphology and structure of TiO_2_ thin films grown by atomic layer deposition. J. Cryst. Growth 1995, 148 (3), 268–275. 10.1016/0022-0248(94)00874-4.

[ref51] Miikkulainen V.; Leskelä M.; Ritala M.; Puurunen R. L. Crystallinity of Inorganic Films Grown by Atomic Layer Deposition: Overview and General Trends. J. Appl. Phys. 2013, 113, 021301 10.1063/1.4757907.

[ref52] Kaindl R.; Homola T.; Rastelli A.; Schwarz A.; Tarre A.; Kopp D.; Coclite A. M.; Görtler M.; Meier B.; Prettenthaler B.; Belegratis M.; Lackner J. M.; Waldhauser W. Atomic Layer Deposition of Oxide Coatings on Porous Metal and Polymer Structures Fabricated by Additive Manufacturing Methods (Laser-Based Powder Bed Fusion, Material Extrusion, Material Jetting). Surf. Interfaces 2022, 34, 102361 10.1016/j.surfin.2022.102361.

[ref53] Biesinger M. C.; Payne B. P.; Grosvenor A. P.; Lau L. W. M.; Gerson A. R.; Smart R. S. C. Resolving Surface Chemical States in XPS Analysis of First Row Transition Metals, Oxides and Hydroxides: Cr, Mn, Fe, Co and Ni. Appl. Surf. Sci. 2011, 257 (7), 2717–2730. 10.1016/j.apsusc.2010.10.051.

[ref54] Fang W.; Xing M.; Zhang J. A New Approach to Prepare Ti^3+^ Self-Doped TiO_2_ via NaBH_4_ Reduction and Hydrochloric Acid Treatment. Appl. Catal., B 2014, 160–161 (1), 240–246. 10.1016/j.apcatb.2014.05.031.

[ref55] Zhao Z.; Zhou Y.; Wan W.; Wang F.; Zhang Q.; Lin Y. Nanoporous TiO_2_/Polyaniline Composite Films with Enhanced Photoelectrochemical Properties. Mater. Lett. 2014, 130, 150–153. 10.1016/j.matlet.2014.05.099.

[ref56] Kehrer M.; Duchoslav J.; Hinterreiter A.; Cobet M.; Mehic A.; Stehrer T.; Stifter D. XPS Investigation on the Reactivity of Surface Imine Groups with TFAA. Plasma Process Polym. 2019, 16 (4), 1800160 10.1002/ppap.201800160.

[ref57] Siimon H.; Aarik J. Thickness Profiles of Thin Films Caused by Secondary Reactions in Flow-Type Atomic Layer Deposition Reactors. J. Phys. D 1997, 30 (12), 1725–1728. 10.1088/0022-3727/30/12/006.

[ref58] Aarik J.; Aidla A.; Mändar H.; Uustare T. Atomic Layer Deposition of Titanium Dioxide from TiCl_4_ and H_2_O: Investigation of Growth Mechanism. Appl. Surf. Sci. 2001, 172 (1–2), 148–158. 10.1016/S0169-4332(00)00842-4.

[ref59] Jakschik S.; Schroeder U.; Hecht T.; Gutsche M.; Seidl H.; Bartha J. W. Crystallization Behavior of Thin ALD-Al_2_O_3_ Films. Thin Solid Films 2003, 425 (1–2), 216–220. 10.1016/S0040-6090(02)01262-2.

[ref60] Kuang C.; Tan P.; Bahadur A.; Iqbal S.; Javed M.; Qamar M. A.; Fayyaz M.; Liu G.; Alzahrani O. M.; Alzahrani E.; Farouk A. E. A. Dye Degradation Study by Incorporating Cu-Doped ZnO Photocatalyst into Polyacrylamide Microgel. J. Mater. Sci. Mater. Electron. 2022, 33 (13), 9930–9940. 10.1007/s10854-022-07984-6.

[ref61] Xiong B.; Loss R. D.; Shields D.; Pawlik T.; Hochreiter R.; Zydney A. L.; Kumar M. Polyacrylamide Degradation and Its Implications in Environmental Systems. NPJ. Clean Water 2018, 1 (1), 1–9. 10.1038/s41545-018-0016-8.

[ref62] Mudassir M. A.; Hussain S. Z.; Kousar S.; Zhang H.; Ansari T. M.; Hussain I. Hyperbranched Polyethylenimine-Tethered Multiple Emulsion-Templated Hierarchically Macroporous Poly(Acrylic Acid)-Al_2_O_3_ Nanocomposite Beads for Water Purification. ACS Appl. Mater. Interfaces 2021, 13 (23), 27400–27410. 10.1021/acsami.1c03922. 34081850

[ref63] Narath S.; Koroth S. K.; Shankar S. S.; George B.; Mutta V.; Wacławek S.; Černík M.; Padil V. V. T.; Varma R. S. Cinnamomum Tamala Leaf Extract Stabilized Zinc Oxide Nanoparticles: A Promising Photocatalyst for Methylene Blue Degradation. Nanomaterials 2021, 11 (6), 1558 10.3390/nano11061558. 34199291 PMC8231933

[ref64] Kovačič S.; Anžlovar A.; Erjavec B.; Kapun G.; Matsko N. B.; Žigon M.; Žagar E.; Pintar A.; Slugovc C. Macroporous ZnO Foams by High Internal Phase Emulsion Technique: Synthesis and Catalytic Activity. ACS Appl. Mater. Interfaces 2014, 6 (21), 19075–19081. 10.1021/am5050482. 25335099

[ref65] Gurevitch I.; Silverstein M. S. Nanoparticle-Based and Organic-Phase-Based AGET ATRP PolyHIPE Synthesis within Pickering HIPEs and Surfactant-Stabilized HIPEs. Macromolecules 2011, 44 (9), 3398–3409. 10.1021/ma200362u.

