# Supplementary material for: Highly Porous Polymer Beads Coated with Nanometer-Thick Metal Oxide Films for Photocatalytic Oxidation of Bisphenol A
Source: ACS Appl Nano Mater. 2023 Oct 24;6(21):20089–98. doi: 10.1021/acsanm.3c03891 (PMC10653210; doi:10.1021/acsanm.3c03891)
Supplement: Supplementary file 1 — an3c03891_si_001.pdf [file an3c03891_si_001.pdf]

Supporting Information

Highly Porous Polymer Beads Coated with Nanometer-Thick Metal Oxide Films for Photocatalytic Oxidation of Bisphenol A

Gergő Ballai[‡a], *Tomaž Kotnik*,[‡b,c] *Matjaž Finšgar*,[d] *Albin Pintar*,[b] *Zoltán Kónya*,[a,e] *András Sápi*,[a] *Sebastijan Kovačič*[b*]

[a]Interdisciplinary Excellence Centre, Department of Applied and Environmental Chemistry, University of Szeged, Rerrich Béla tér 1, H-6720 Szeged, Hungary

[b]Department of Inorganic Chemistry and Technology, National Institute of Chemistry, Hajdrihova 19, SI-1001 Ljubljana, Slovenia; *sebastijan.kovacic@ki.si

[c]Faculty of Chemistry and Chemical Technology, University of Ljubljana, Večna Pot 113, 1000 Ljubljana, Slovenia

[d]University of Maribor, Faculty of Chemistry and Chemical Engineering, Smetanova 17, SI-2000 Maribor, Slovenia

[e]MTA-SZTE Reaction Kinetics and Surface Chemistry Research Group, Rerrich Béla tér 1, H-6720 Szeged, Hungary

‡ G.B. and T.K. contributed equally to this paper.

Table S1. Typical recipe for PAAM-based PH synthesis.

	**PAAM**
**Aqueous External Phase, wt.%**	
H_2O	15.19
AAm	3.11
MBAAm	0.63
F108	1.46
APS	0.37
TEMED	0.19
Total	20.95
**Organic Internal Phase, wt.%**	
Toluene	79.05
Total	79.05

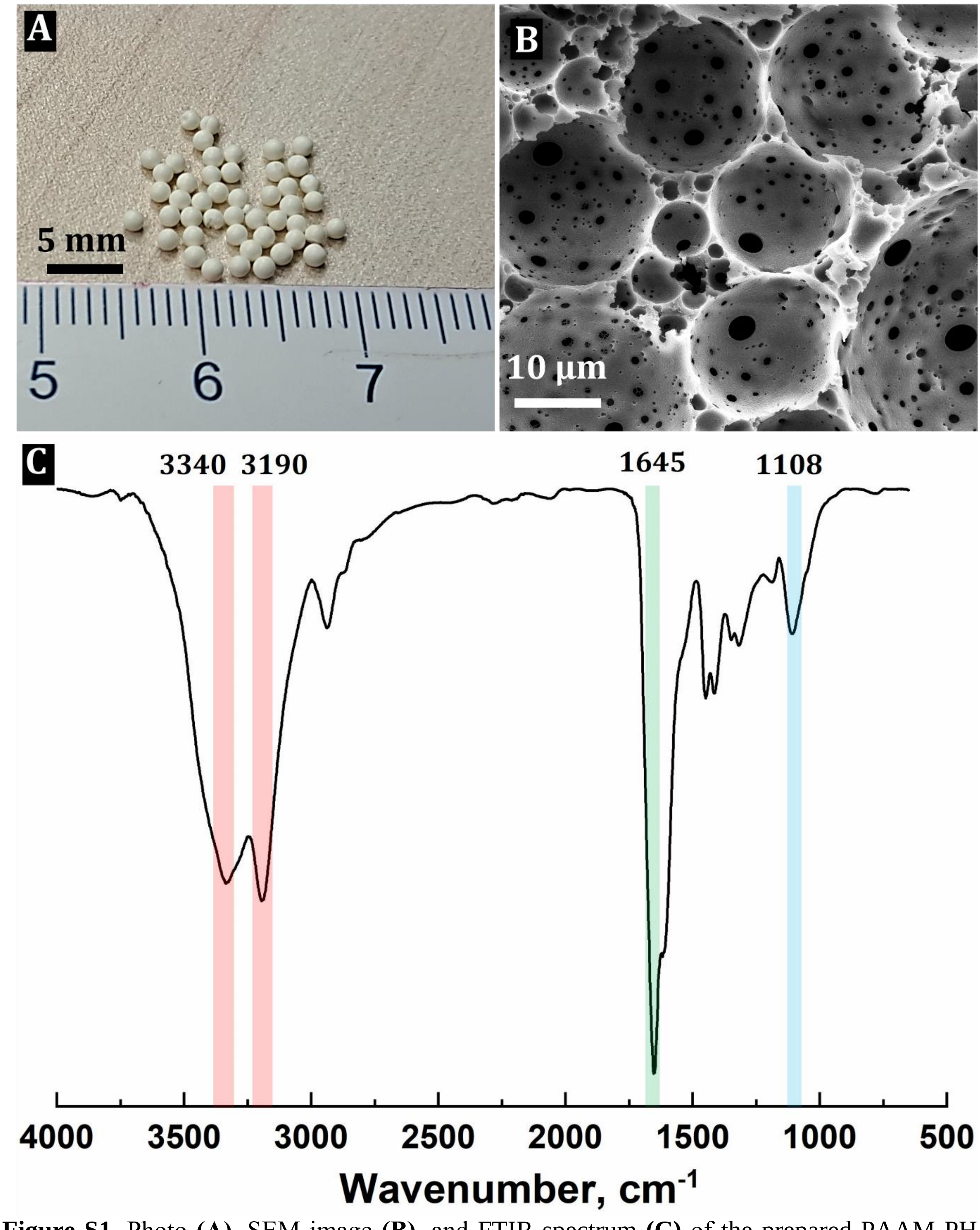

Figure S1. Photo **(A)**, SEM image **(B),** and FTIR spectrum **(C)** of the prepared PAAM PH beads.

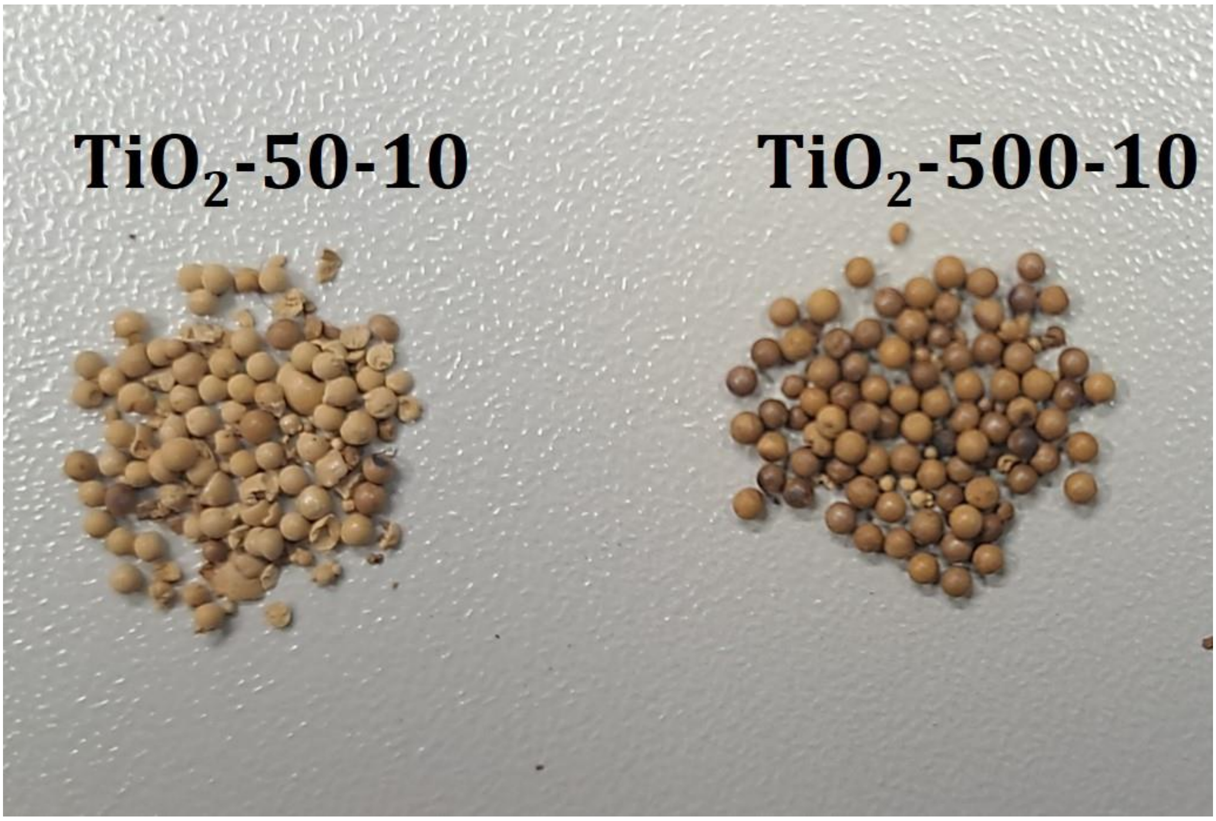

Figure S2. Different colours of the beads after 50 and 500 ALD cycles programme.

TiO_2-Pickering PH beads

TiO_2-PAAM-PolyHIPE beads were prepared in the same way as pure PAAM PH beads, i.e., by O/W/O sedimentation polymerization. First, oil-in-water (O/W) HIPEs were prepared using AAM (0.86 g), MBAM (0.152 g), APS (0.1 g), TEMED (0.08 mL), surfactant (Pluronic F-108), and TiO_2 nanoparticles (0.1 g) as the (continuous) water phase, to which toluene (20 mL) was added dropwise. Subsequently, this O/W Pickering HIPE was injected dropwise into the second continuous phase (paraffin oil), also containing TMEDA, using a syringe with a needle (external diameter 0.8 mm). 40 mL of paraffin oil was charged into a 50 mL graduated cylinder, degassed in an ultrasonic bath, and then heated to 85 ºC in a water bath. After adding all the O/W HIPE dropwise, the graduated cylinder was left in the water bath for 1 hour and then transferred to an oven at 50 ºC for 24 hours to complete polymerization. After polymerization, the beads were collected and cleaned with ethanol for 24 hours and ether for 24 hours in a Soxhlet apparatus and dried in vacuo.

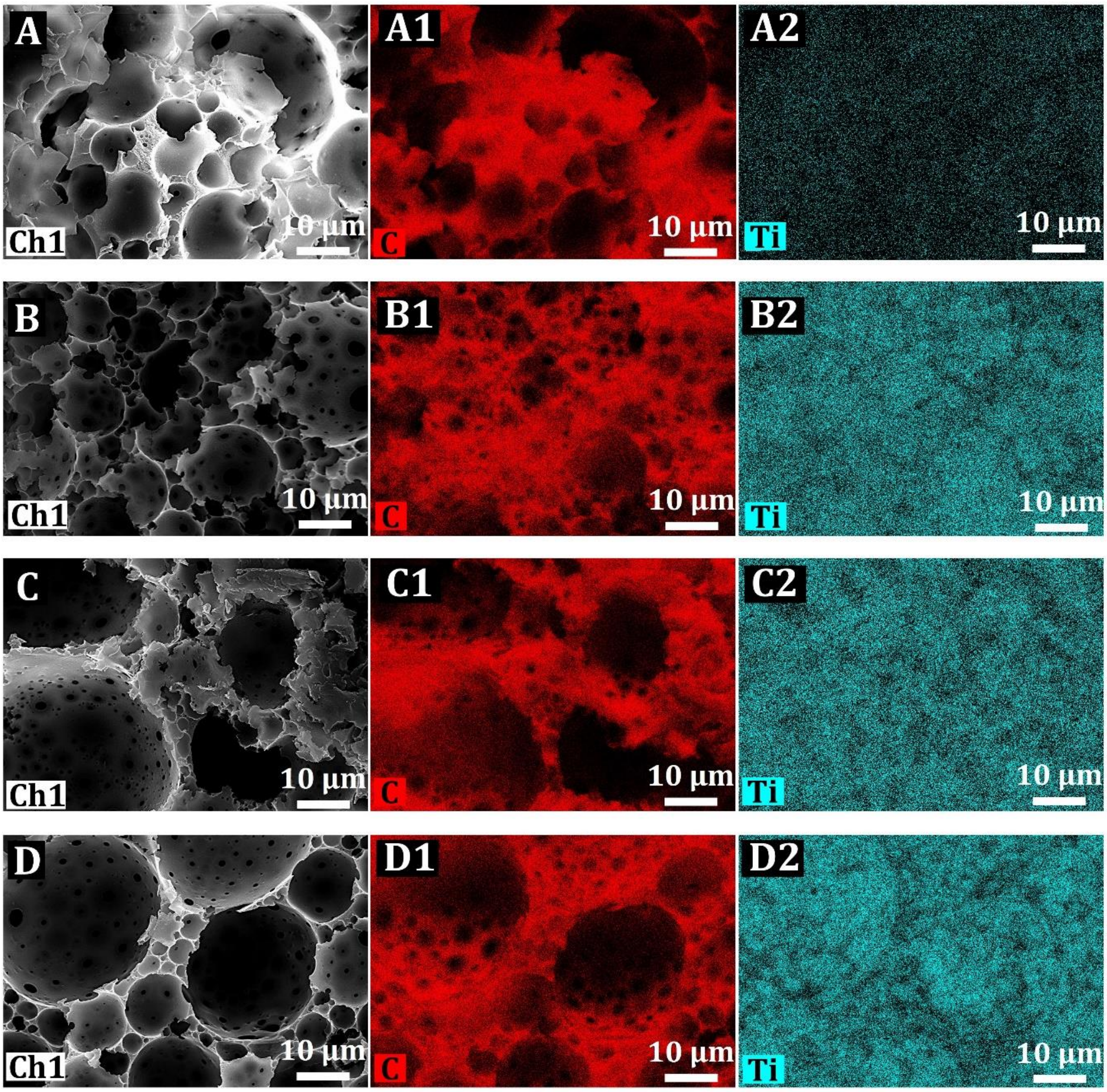

Figure S3. SEM-EDX elemental mapping for Ti atom of TiO_2-50-10 **(A)**, TiO_2-100-10 **(B)**, TiO_2-250-10 **(C),** and TiO_2-500-10 PH beads **(D)**.

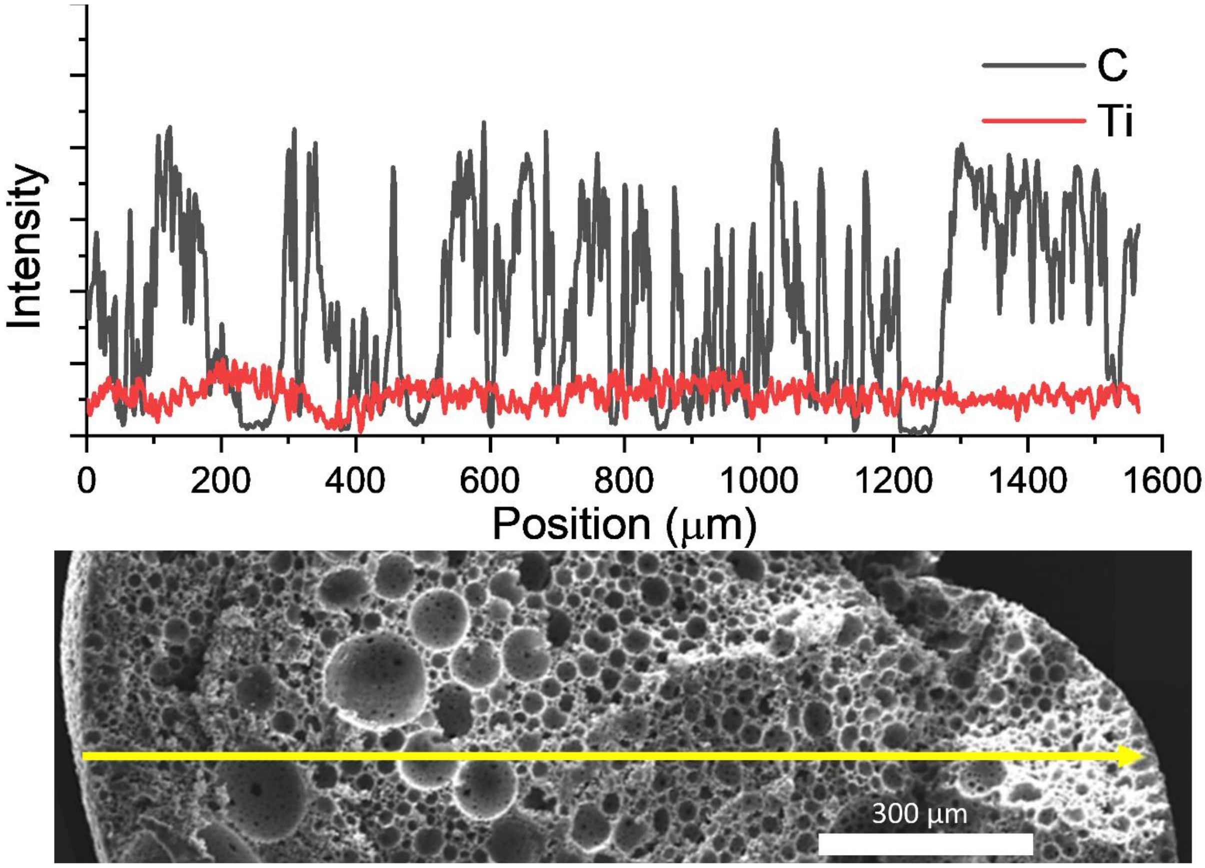

Figure S4. SEM-EDS line profile analysis of TiO_2-250-10 showing a distribution of Ti and C on the surface of the voids over the entire bead diameter.

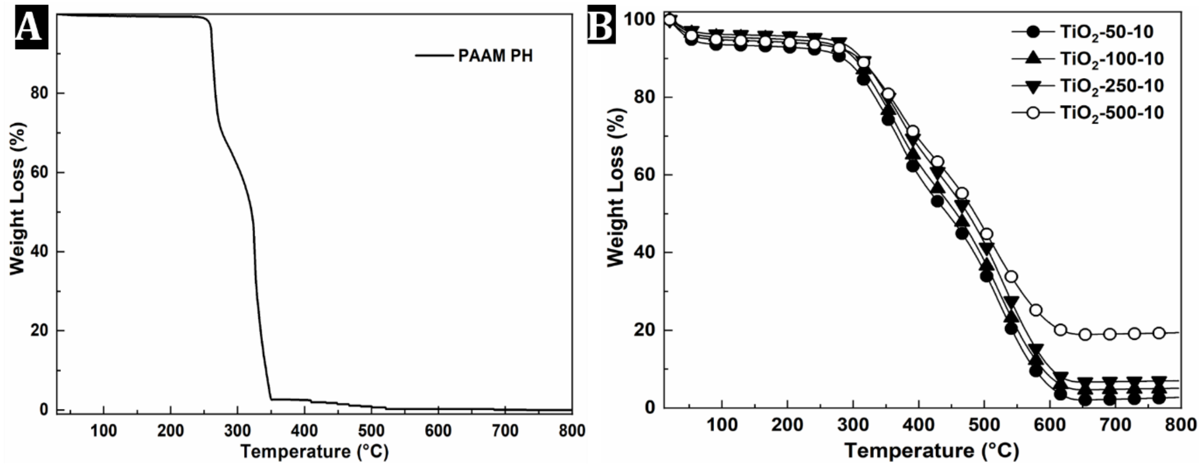

Figure S5. TGA profile for the net PAAM PH sample **(A)** and TGA profiles of TiO_2-X-10 sample series **(B)** obtained in an oxygen atmosphere.

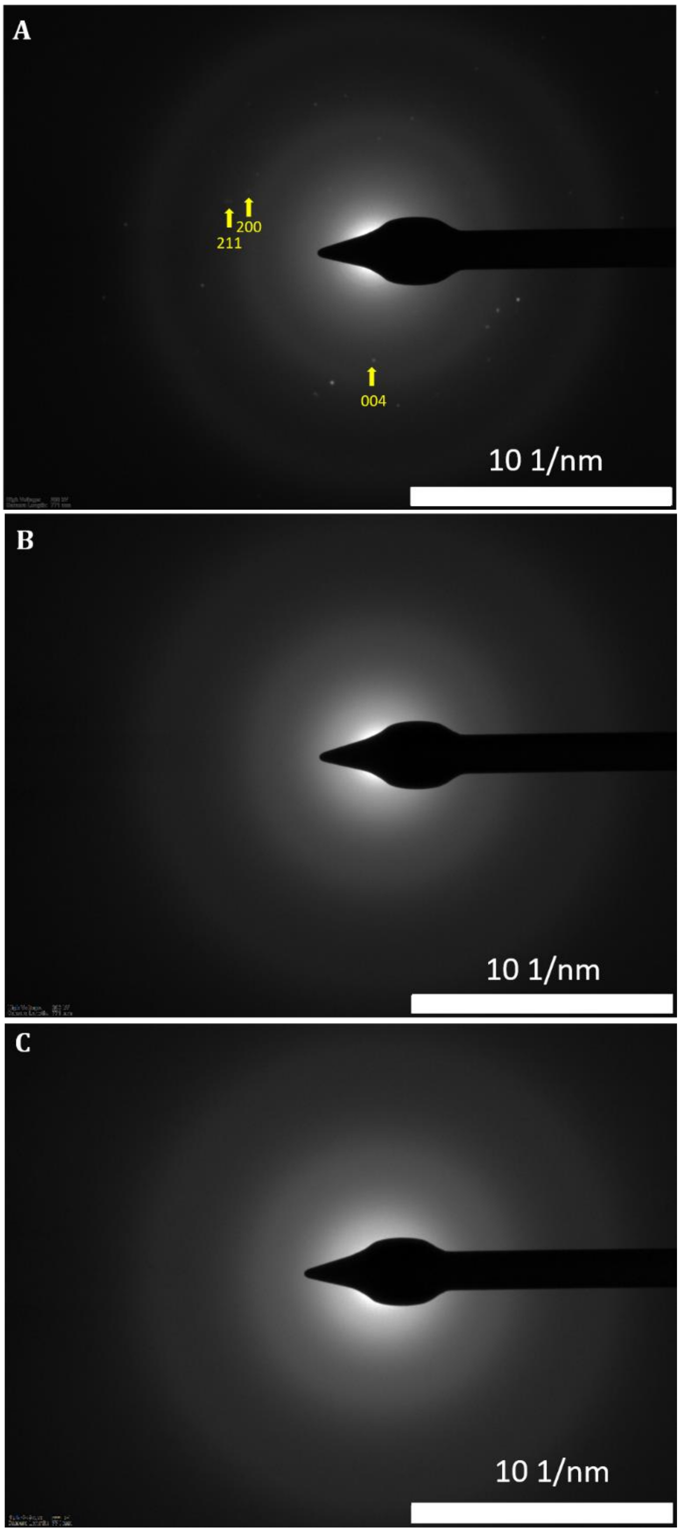

Figure S6. SAED patterns of TiO_2-250-10 **(A)**, TiO_2-100-10 **(B)**, and TiO_2-50-10 **(C)**.

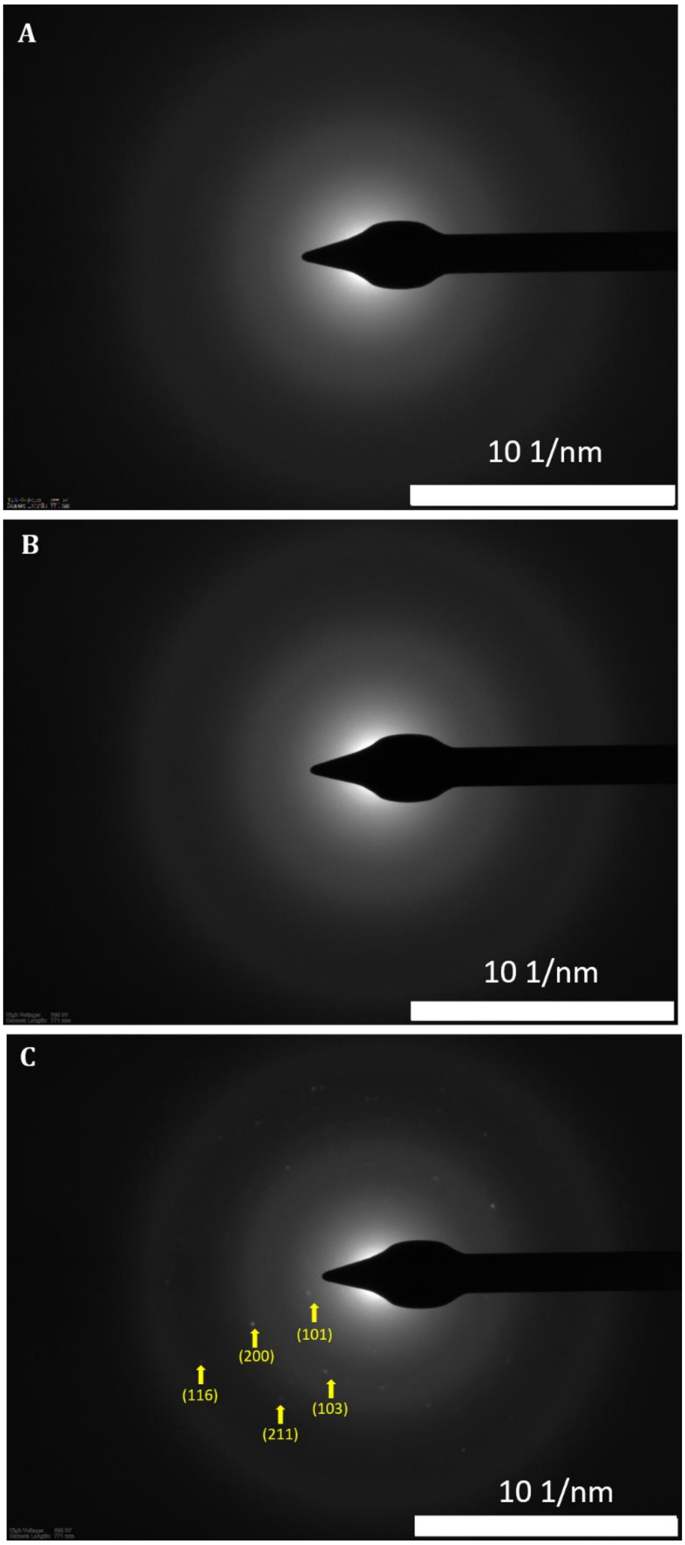

Figure S7. SAED patterns of TiO_2-50-30 **(A)**, TiO_2-50-60 **(B)**, and TiO_2-50-100 **(C)**.

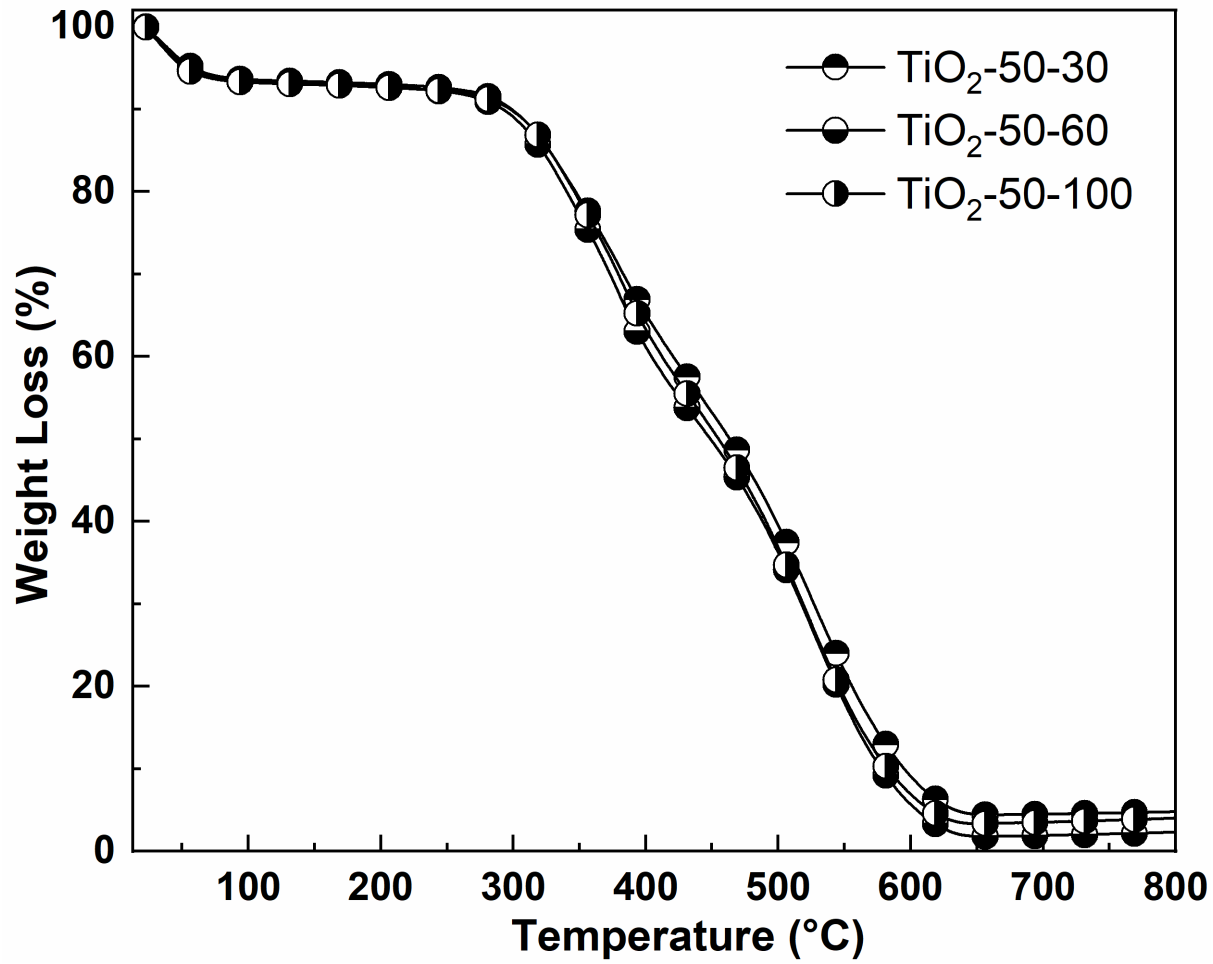

Figure S8. TGA profiles of TiO_2-50-X sample series obtained in oxygen atmosphere.

X-ray photoelectron spectroscopy (XPS)

XPS measurements were performed using a Supra+ device (Kratos, Manchester, UK) equipped with an Al K_α excitation source. During the measurements, the charge neutralizer was on. Spectra were acquired at a 90° take-off angle (the angle with respect to the sample holder). Spectra were corrected using a C-C/C-H peak in the C 1s spectra at a binding energy of 284.8 eV. High-resolution and survey spectra were acquired at 20 eV and 160 eV pass energy, respectively. The base pressure in the analysis chamber was $2 \cdot 10^{-9}$ torr. Spectra acquisition and data processing were performed using ESCApe 1.5 software (Kratos). Samples were attached to the sample holder using carbon tape. Sputtering was performed with a gas cluster ion beam using 10 keV Ar_{1000}^+ and 5 keV Ar^+. The sputtering cycle for 10 keV Ar_{1000}^+ and 5 keV Ar^+ were 30 s and 120 s, respectively.

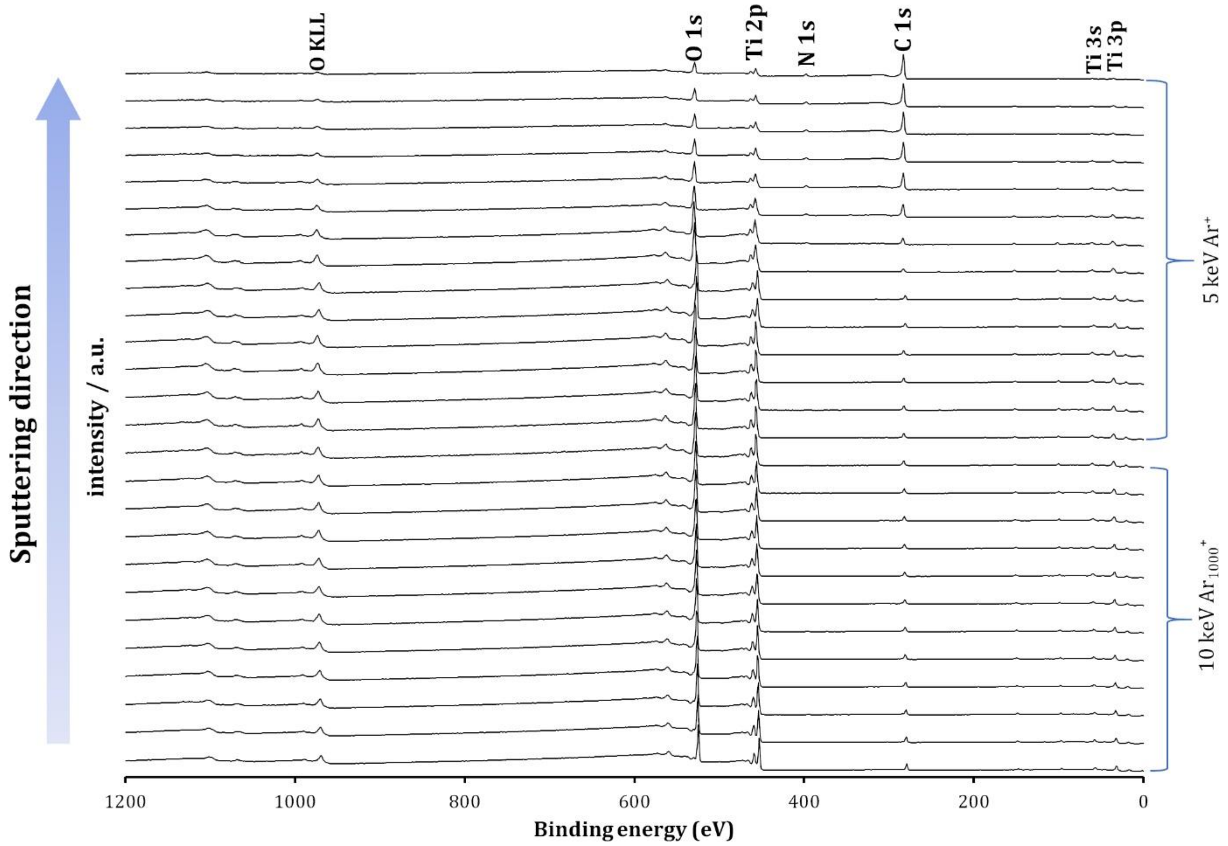

Figure S9. The survey spectra of TiO_2-250-10 sample show the presence of Ti 2p, Ti 3s, Ti 3p, O 1s, N 1s, and C 1s XPS signals, XPS-induced O KLL Auger signal.

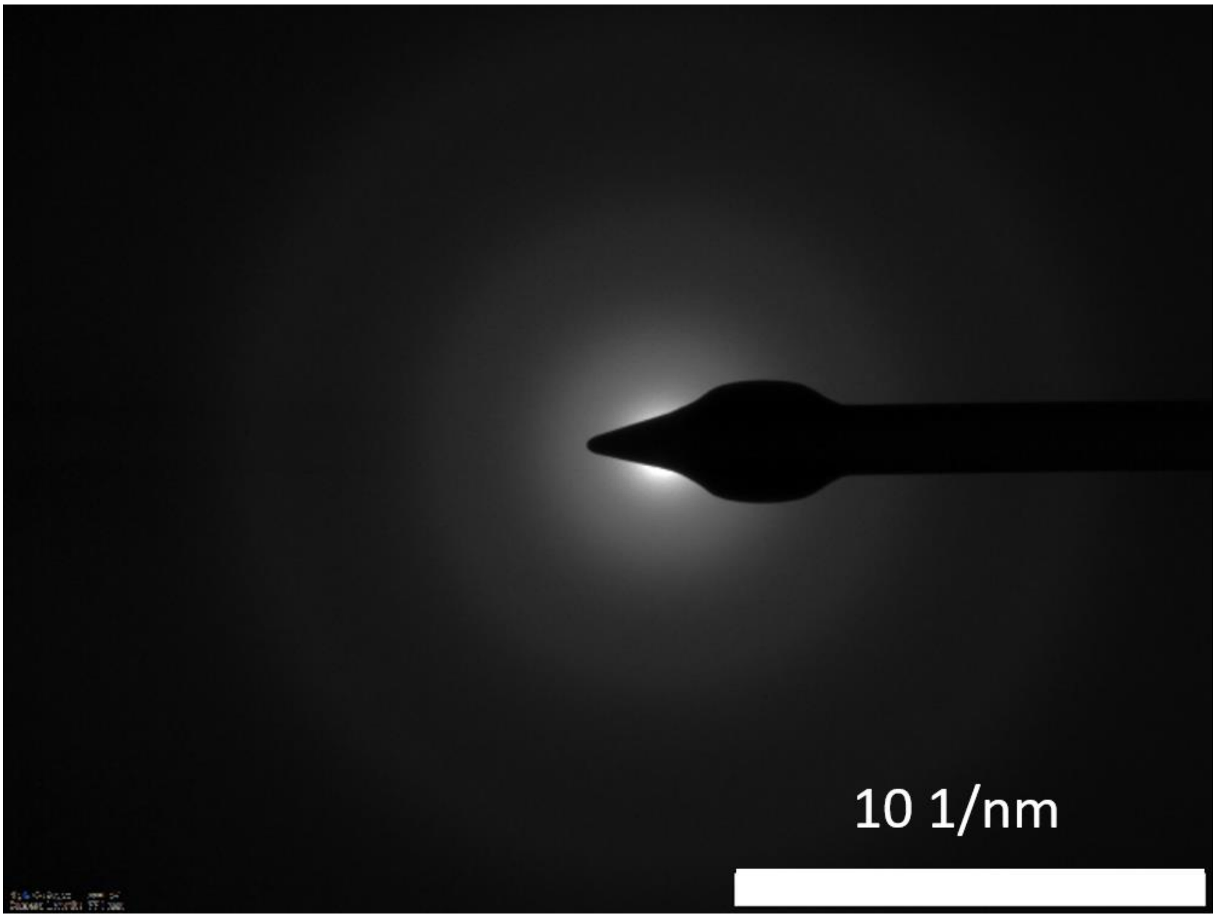

Figure S10. SAED pattern of Al_2O_3-250-10 sample.

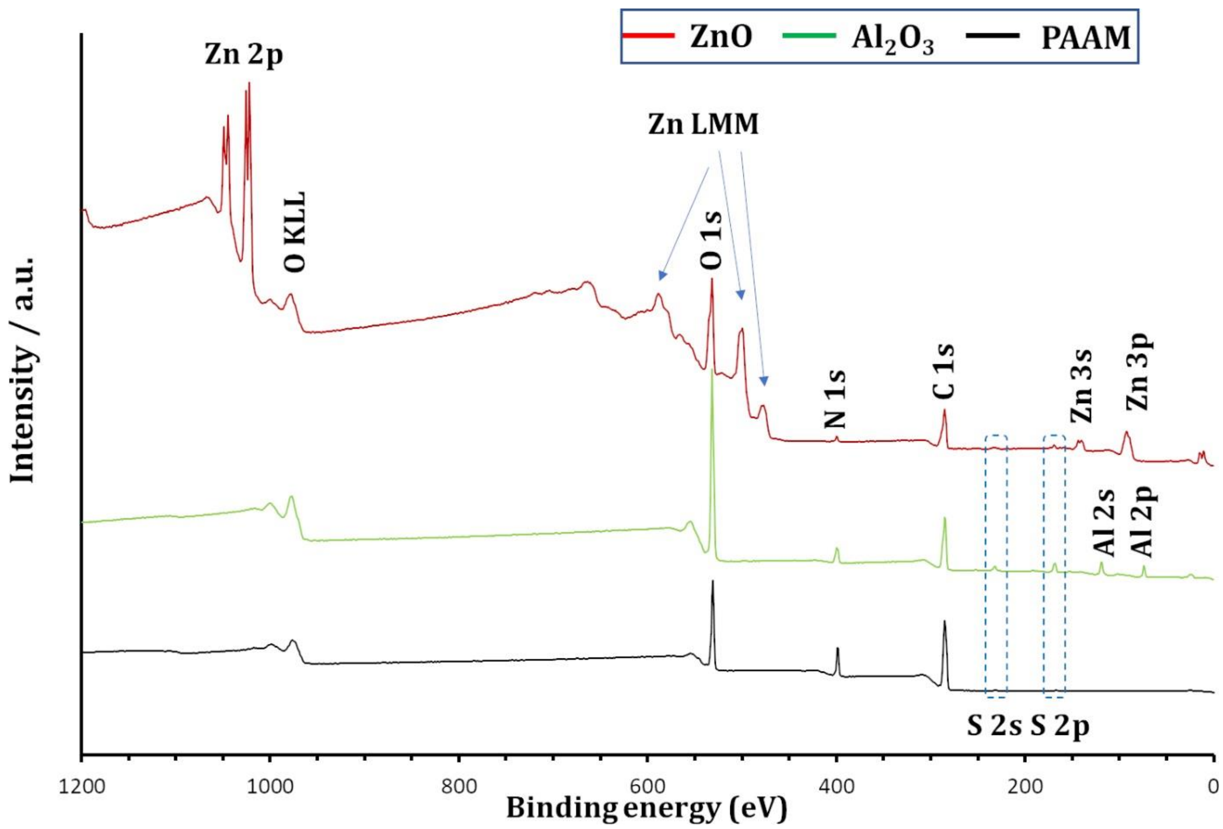

Figure S11. Survey spectra measured for ZnO, Al_2O_3, and PAAM.

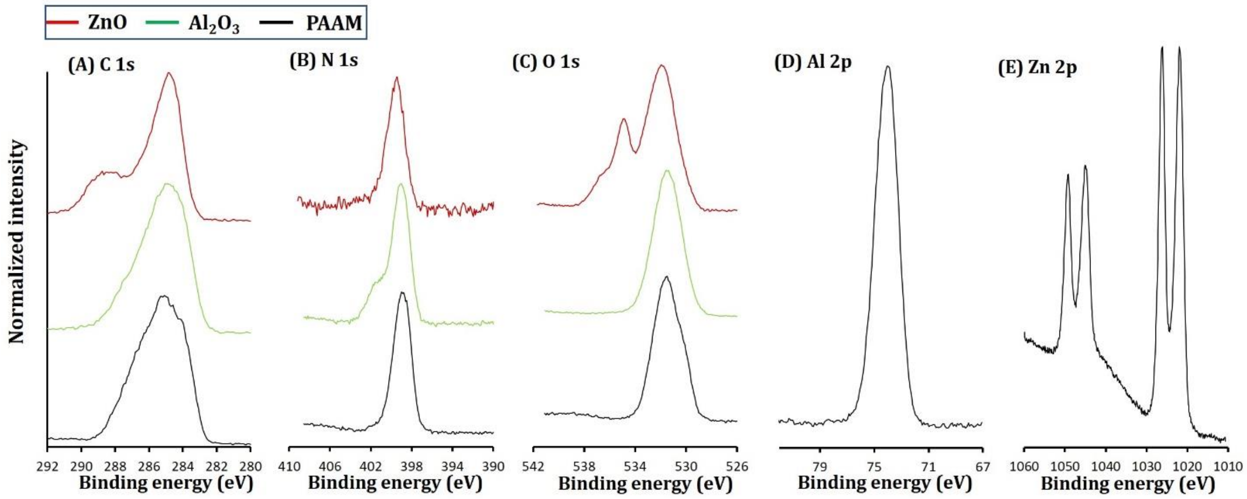

Figure S12. High-resolution XPS spectra for Al_2O_3- and ZnO-250-10 samples, and PAAM: C 1s **(A)**, N 1s **(B)**, O 1s **(C)**, Al 2p **(D)**, and Zn 2p **(E)**.

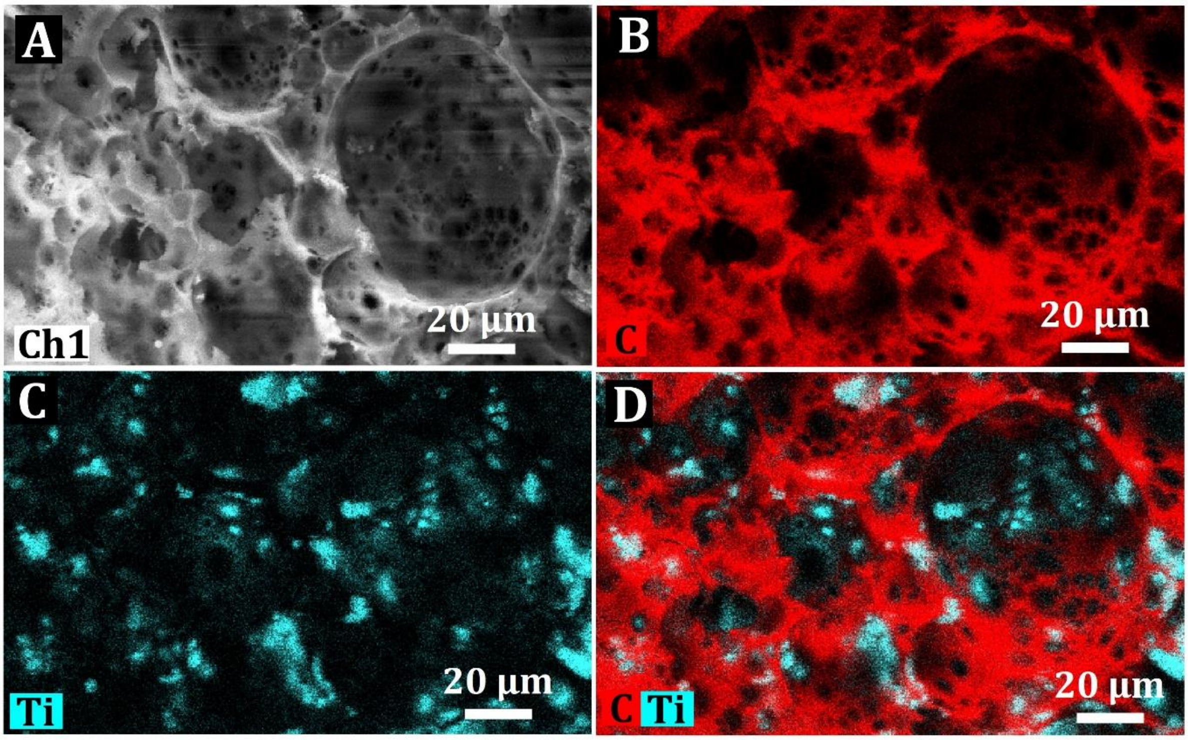

Figure S13. SEM-EDX elemental mapping in Pickerign PH beads.

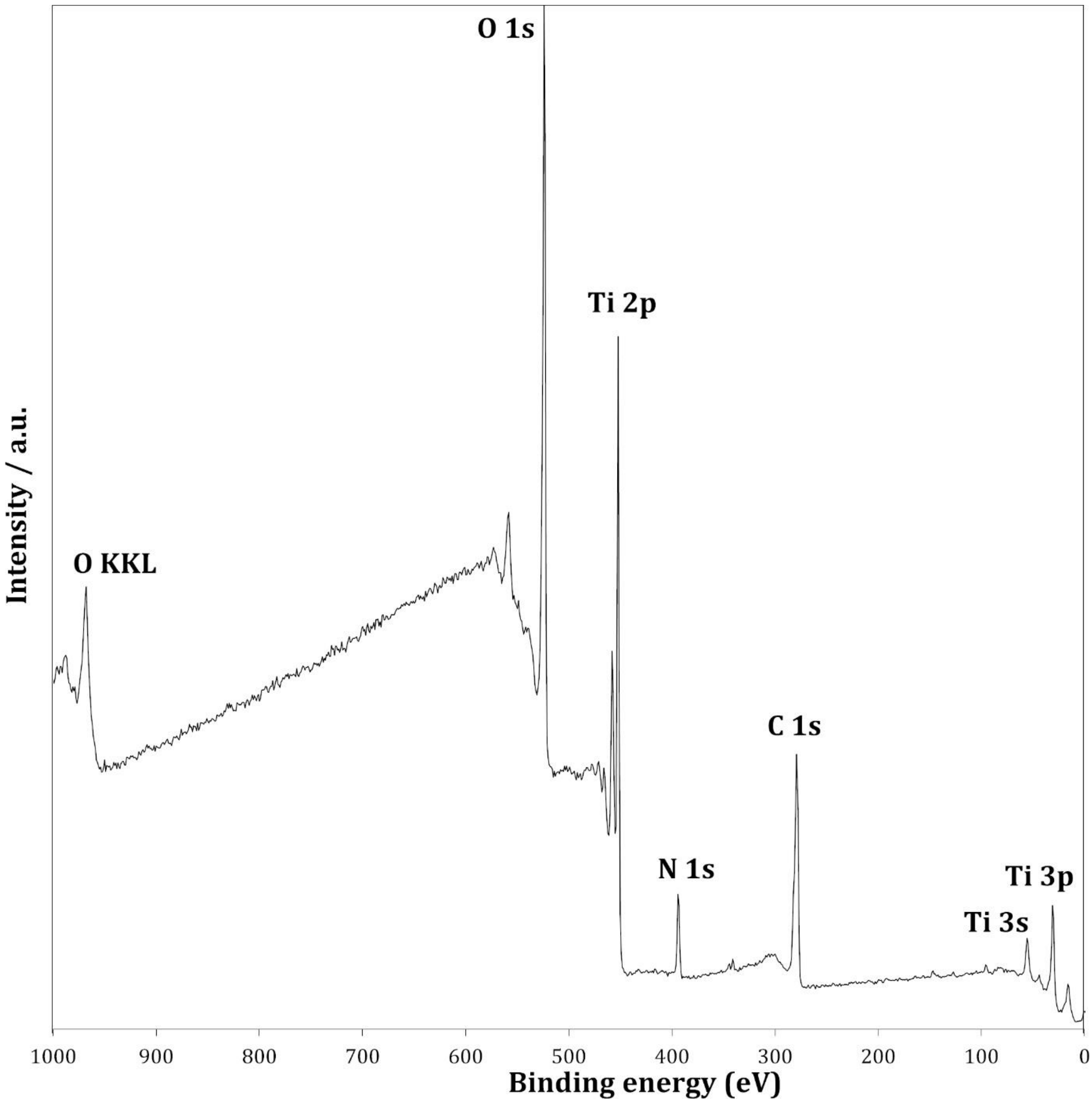

Figure S14. Survey spectra of TiO_2-250-10 sample after five consecutive photocatalytic cycles.

Photocatalytic oxidation experiments

Photocatalytic experiments were performed in a batch slurry reactor (Lenz, Wertheim, Germany, model LF60, 250 mL) at 20 °C (Julabo, Seelbach, Germany, model F25/ME) and atmospheric pressure. Throughout the oxidation run, the aqueous solution (ultrapure water, 18.2 MΩ cm) of bisphenol A (BPA, c_0=10 mg/L, 100 ml, Aldrich) was purged with purified air (25 L/h) and magnetically stirred (400 rpm). Suspended in this solution were 140 mg PH nanocomposite beads corresponding to the 7 weight percent of TiO_2. The amount of TiO_2 was the same for both ALD-derived and Pickering PH beads. The equilibrium of the sorption process was established during the 30 minutes that the aqueous suspension was kept in the dark ("dark phase"). Then, the suspension was illuminated with a UVA Hg lamp (Philips, Amsterdam, The Netherlands, 150 W, maximum at λ=365 nm) located in a water-cooled quartz jacket immersed vertically in the center of the batch slurry reactor.

During the photocatalytic runs, representative 1.5 mL samples were withdrawn in 5 to 30 min intervals from the reactor and immediately filtered through a 0.2 µm membrane filter before being analyzed with an HPLC instrument (Thermo Scientific, Waltham, MA, USA, model Spectra) to determine temporal BPA conversions. The isocratic analytical mode using a 100 mm × 4.6 mm BDS Hypersil C18 2.4 µm column thermostated at 30 °C was used to perform HPLC measurements. The flow rate of the mobile phase (70% methanol (Merck, Darmstadt, Germany) and 30% ultrapure water) was 0.5 mL/min, and UV detection was conducted at λ=210 nm.
